# Proteome‐wide profiling reveals dysregulated molecular features and accelerated aging in osteoporosis: A 9.8‐year prospective study

**DOI:** 10.1111/acel.14035

**Published:** 2023-11-16

**Authors:** Jinjian Xu, Xue Cai, Zelei Miao, Yan Yan, Danyu Chen, Zhen‐xiao Yang, Liang Yue, Wei Hu, Laibao Zhuo, Jia‐ting Wang, Zhangzhi Xue, Yuanqing Fu, Ying Xu, Ju‐Sheng Zheng, Tiannan Guo, Yu‐ming Chen

**Affiliations:** ^1^ Department of Epidemiology, Guangdong Provincial Key Laboratory of Food, Nutrition and Health, School of Public Health Sun Yat‐sen University Guangzhou China; ^2^ School of Life Sciences Westlake University Hangzhou China; ^3^ Westlake Center for Intelligent Proteomics, Westlake Laboratory of Life Sciences and Biomedicine Hangzhou China; ^4^ Shenzhen Bao'an Center for Chronic Diseases Control Shenzhen China

**Keywords:** biological age, longitudinal study, osteoporosis, proteome‐wide study

## Abstract

The role of circulatory proteomics in osteoporosis is unclear. Proteome‐wide profiling holds the potential to offer mechanistic insights into osteoporosis. Serum proteome with 413 proteins was profiled by liquid chromatography–tandem mass spectrometry (LC–MS/MS) at baseline, and the 2nd, and 3rd follow‐ups (7704 person‐tests) in the prospective Chinese cohorts with 9.8 follow‐up years: discovery cohort (*n* = 1785) and internal validation cohort (*n* = 1630). Bone mineral density (BMD) was measured using dual‐energy X‐ray absorptiometry (DXA) at follow‐ups 1 through 3 at lumbar spine (LS) and femoral neck (FN). We used the Light Gradient Boosting Machine (LightGBM) to identify the osteoporosis (OP)‐related proteomic features. The relationships between serum proteins and BMD in the two cohorts were estimated by linear mixed‐effects model (LMM). Meta‐analysis was then performed to explore the combined associations. We identified 53 proteins associated with osteoporosis using LightGBM, and a meta‐analysis showed that 22 of these proteins illuminated a significant correlation with BMD (*p* < 0.05). The most common proteins among them were PHLD, SAMP, PEDF, HPTR, APOA1, SHBG, CO6, A2MG, CBPN, RAIN APOD, and THBG. The identified proteins were used to generate the biological age (BA) of bone. Each 1 SD‐year increase in KDM‐Proage was associated with higher risk of LS‐OP (hazard ratio [HR], 1.25; 95% CI, 1.14–1.36, *p* = 4.96 × 10^−06^), and FN‐OP (HR, 1.13; 95% CI, 1.02–1.23, *p* = 9.71 × 10^−03^). The findings uncovered that the apolipoproteins, zymoproteins, complements, and binding proteins presented new mechanistic insights into osteoporosis. Serum proteomics could be a crucial indicator for evaluating bone aging.

## Graphical abstract

Serum proteomics offers potential insight into accelerated aging in osteoporosis. Osteoporosis is commonly referred to as an aging disorder characterized by decreased bone mineral density (BMD) and an elevated risk of fractures. Using longitudinal serum proteomics analyses with 413 protein species covering various protein classes in a population‐based study, we found that the proteins of apolipoproteins, zymoprotein, coagulation, immunoglobulins, complement, and binding proteins may be the potential therapeutic targets for osteoporosis. Serum proteomics could be a crucial indicator for evaluating bone aging.

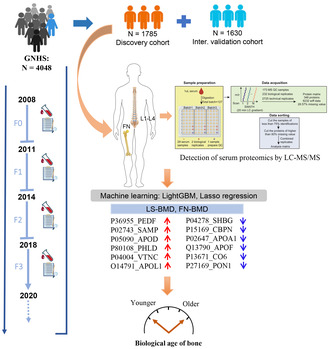

**Abbreviations**
BA: biological age
BH: Benjamini–Hochberg
BIC: Bayesian information criterion
BMD: bone mineral density
BMI: body mass index
CA: chronological age
CIs: confidence intervals
DBP: diastolic blood pressure
DXA: dual‐energy X‐ray absorptiometry
FDR: false discovery rates
FN: femoral neck
GNHS: Guangzhou Nutrition and Health Study
GLM: generalized linear model
GWAS: Genome Wide Association Study
HC: hip circumference
HDL‐C: high‐density lipoprotein cholesterol
IVW: Inverse variance weighted
KDM: Klemera and Doudal algorithm
LASSO: least absolute shrinkage and selection operator
LC‐MS/MS: liquid chromatography‐tandem mass spectrometry
LCTM: latent class trajectory model
LDL‐C: low‐density lipoprotein cholesterol
LightGBM: Light Gradient Boostin Machine
LMM: linear mixed‐effects model
LS: lumbar spine
MR: Mendelian randomization
OP: osteoporosis
PCA: principal component analysis
PRS: protein risk scores
RCS: restricted cubic spline
ROC: receiver operating characteristic
SBP: systolic blood pressure
SD: standard deviations
SHAP: Shapley Additive exPlanations
SNP: single nucleotide polymorphism
TC: total cholesterol
TG: triglyceride
WC: waist circumference
WHR: waist‐to‐hip ratio

## 1 INTRODUCTION

Osteoporosis (OP) is a common skeletal disorder characterized by decreased bone mineral density (BMD) and an elevated risk of fractures (Qaseem & Wilt, 2021). It is estimated that more than 25% of individuals aged 50 and above worldwide had osteoporosis in 2017 (Roux & Briot, 2018), while the prevalence of osteoporosis among Chinese individuals aged 65 and above was approximately 32% in 2019 (Wang et al., 2021). Osteoporotic fractures represent a significant source of disability and economic burden, particularly among middle‐aged and elderly populations as life expectancy continues to rise (Crandall et al., 2020).

The vertebral bodies, which include trabecular bone, make up the majority of the lumbar spine (LS) (Lorentzon, 2020). Trabecular bone consists of small, dense bone trabeculae interconnected by numerous small spaces, forming a spongy structure (Nethander et al., 2020). The femoral neck (FN), on the contrary, is made up of tightly packed cortical bone tissue (Samelson et al., 2019). The LS generally exhibits relatively lower overall BMD due to its predominant trabecular bone. Conversely, the FN bone possesses higher BMD. Osteoporosis at LS and FN often involves an increase in bone resorption, wherein osteoclasts, the bone‐resorbing cells, become excessively active (Wang, You, et al., 2020). The osteoblasts are responsible for bone formation, the reduced functionality in osteoblasts leads to a deceleration in bone remodeling (Wu et al., 2020).

Circulating proteomics are impressionable codes for the biological processes in multiple tissues and organs (Geyer et al., 2019), which have been recognized as a sensitive and accurate fingerprint for human aging (Sathyan, Ayers, Gao, Milman, et al., 2020) and related diseases (Bhardwaj et al., 2020; Ngo et al., 2021). Previous studies have uncovered the existence of aging proteins that can precisely predict human frailty (Sathyan, Ayers, Gao, Milman, et al., 2020) and chronological age (CA) (Sathyan, Ayers, Gao, Weiss, et al., 2020) and were significantly associated with all‐cause mortality in adults (Sathyan, Ayers, Gao, Weiss, et al., 2020). Plasma protein biomarkers have been shown to accurately estimate the severity of Alzheimer's disease (AD) (Jiang et al., 2022), evaluate the risk of type 2 diabetes (T2DM) (Ngo et al., 2021) and cardiometabolic diseases (Ritchie et al., 2021) in elderly adults. Osteoporosis as a parallel degeneration with aging, however, it is largely unknown which and to what extent the circulatory proteins might affect bone loss in humans due to the scarcity of evidence. The limited studies have reported that the majority of the identified serum proteins associated with bone loss were immunoglobulins, complement proteins, cytoskeletal proteins, coagulation factors, and various enzymes using the technologies of label‐free liquid chromatography‐mass spectrometry (LC–MS) and the multidimensional approach coupling liquid chromatography, ion‐mobility separation, and mass spectrometry (LC‐IMS‐MS) (Al‐Ansari et al., 2022; Nielson et al., 2017).

Using proteomic techniques, numerous critical proteins have been identified in relation to BMD and osteoporosis. The study in postmenopausal osteoporosis indicated that the expression of protein lysozyme C (P61626) was negatively related to BMD, while the proteins glucosidase (A0A024R592) and protein disulfide‐isomerase A5 (Q14554) were positively associated with BMD (Huang et al., 2020). Furthermore, the prospective study in elderly males (*n* = 2473, 4.6 follow‐up years) has uncovered 20 proteins that are enriched in complement activation and innate immune response pathways and are associated with BMD loss. Some proteins were associated with hip fractures (*n*/cases = 2473/124) (such as CD14, SHBG, CO7, CO9, CFAD, B2MG, and TIMP1) (Nielson et al., 2017). Collectively, the results suggested that participants with low BMD have a different proteomic profile or signature. Circulatory protein alterations may play an important role in the pathogenesis of bone loss, and they may act as novel biomarkers and targets of therapeutic agents for osteoporosis (Zeng et al., 2016; Zhang et al., 2016). It is important to note that the proteomics‐based researches for bone health in available studies has been restricted by the small sample size (Zhang et al., 2016), limited detection technology for proteins (Martínez‐Aguilar et al., 2019), and single time‐point measurements (Al‐Ansari et al., 2022). Additionally, previous proteomics studies provided promising evidence of mechanisms and therapeutic targets for aging‐related diseases (Ferrannini et al., 2020; Ngo et al., 2021), but few studies focused on the causal relationship using Mendelian randomization analysis (Chen et al., 2022). The proteomics‐based studies have offered promising avenues for exploring the relationship between serum proteins and osteoporosis, and more comprehensive investigations may shed light on the underlying mechanisms and potential biomarkers of bone loss.

Hence, we conducted a prospective study to investigate the associations of serum proteomics with OP based on the Guangzhou Nutrition and Health Study (GNHS) 2008–2019. The causal association between plasma protein and BMD was further validated using two‐sample Mendelian randomization analysis in external East Asian and European populations. We used BMD‐proteins and the Klemera and Doubal mathematical model (KDM) to generate biological ages (BAs) in order to explore the accelerated aging of bone.

## 2 RESULTS

### 2.1 Overview of the study

**FIGURE 1:**
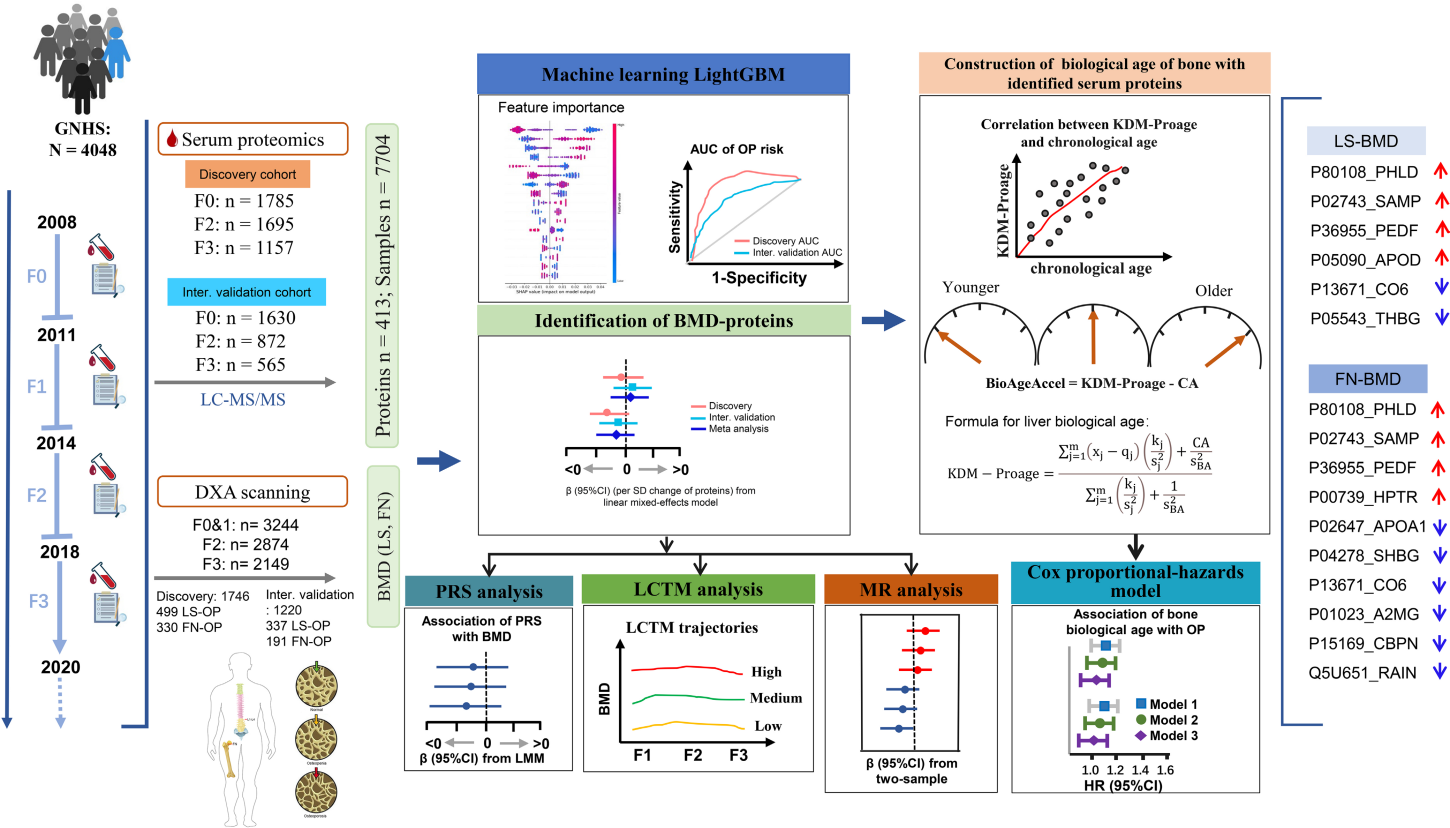
Overview of the study cohort and design. This study included 3244 participants from the Guangzhou Nutrition and Health Study (GNHS), followed up every 3 years from 2008 to 2019. Serum proteomics was profiled using a liquid chromatography–tandem mass spectrometry (LC–MS/MS) at baseline (F0) and follow‐ups (F) 2 and 3. Bone mineral density (BMD) was measured at F1 through F3 at the lumbar spine (LS) and femoral neck (FN). The LightGBM algorithm was used to identified OP‐proteins from 1746 samples including 314 serum proteins in the discovery cohort (training and testing datasets), and the model was validated in the internal validation cohort with 1220 samples. The associations between baseline serum proteins and BMDs at the lumbar spine (LS) and femoral neck (FN) were estimated, respectively, by linear mixed‐effects model (LMM) in the discovery and internal validation cohorts. The dependent variables were the BMD levels at three follow‐ups. The combined effects of proteins on BMDs from the discovery and internal validation cohorts were assessed using the random‐effects meta‐analysis. We constructed protein risk scores (PRS) based on the protein biomarkers significantly related to the site‐specific bone outcomes to evaluate the joint “effect” on the bone outcomes. We compared protein means by the site‐specific BMD trajectories identified from the latent class trajectory model (LCTM). In addition, Mendelian randomization analyses were performed to validate the potential causal effects of the serum proteins on BMD. We generated the biological ages (BAs) with BMD‐proteins by the Klemera and Doubal method (KDM) to investigate the aging rates of bone. BMD, bone mineral density; DXA, dual‐energy X‐ray absorptiometry; FN, femoral neck; GNHS, Guangzhou Nutrition and Health Study; KDM‐Proage, KDM protein age; LC–MS/MS, liquid chromatography–tandem mass spectrometry; LCTM, latent class trajectory model; LightGBM, Light Gradient Boosting Machine; LMM, linear mixed‐effects model; LS, lumbar spine; MR, Mendelian randomization; PRS, protein risk score.

The overview of study design was presented in Figure 1. This study included 3244 participants from the Guangzhou Nutrition and Health Study (GNHS) (Ling et al., 2023), followed up every 3 years from 2008 to 2019. We profiled 413 serum proteins from ~20,000 proteomes at baseline (F0) and follow‐ups (F) 2 and 3 and determined bone mineral density (BMD) at F1 to F3 at lumbar spine (LS) and femoral neck (FN). First, we identified baseline protein biomarkers for osteoporosis by LightGBM in the discovery and internal validation cohorts. Then, the associations between baseline serum proteins and BMDs were estimated by linear mixed‐effects model (LMM) in the discovery and internal validation cohorts. The combined effects of proteins on BMDs from the discovery and internal validation cohorts were assessed using the meta‐analysis. The significant proteins were then used to construct protein risk scores (PRS) to evaluate the joint “effect” on the bone outcomes. We further compared protein means by the site‐specific BMD trajectories identified from the latent class trajectory model (LCTM). In addition, Mendelian randomization analyses were performed to validate the potential causal effects of plasma proteins on BMD. We generated biological ages (BAs) with BMD‐proteins by the Klemera and Doubal model (KDM) to investigate the bone aging.

### 2.2 Participant characteristics

The baseline characteristics in the discovery (*n* = 1785) and internal validation (*n* = 1630) cohorts were presented in Table 1. The characteristics of participants were assessed at each visit. The mean (SD) of age and BMI of participants in baseline were 57.5 (5.1) years and 23.1 (3.0) kg/m^2^, with 72% were females. The mean (SD) follow‐up years in this study was 9.8 (0.7) across all study visits (Table S1). The osteoporosis events during the follow‐up years were 836 at LS and 521 at FN. The baseline characteristics between controls and osteoporosis group in the discovery and internal validation cohorts were shown in Tables S2,S3. Over the course of three follow‐up visits during the 9.8 follow‐up years, the LS‐BMD decreased significantly (Figure S1).

**TABLE 1:** The baseline characteristics from the discovery and internal validation participants.

Characteristics	Overall	Discovery	Internal validation	*p* Value
*N*	3415	1785	1630	
Age (years)	57.48 (5.12)	57.50 (4.86)	57.46 (5.39)	0.200
Sex, *n* (%)				
Female	1945 (72%)	1014 (71%)	931 (72%)	0.500
Male	765 (28%)	409 (29%)	356 (28%)	
Physical activity (MET•hours/d)	41.41 (17.38)	41.54 (17.48)	41.28 (17.27)	0.500
Body mass index (kg/m^2^)	23.28 (3.15)	23.38 (3.09)	23.17 (3.21)	0.073
Waist circumference (cm)	82.23 (9.52)	82.66 (9.88)	81.76 (9.09)	0.005
Hip circumference (cm)	92.20 (10.82)	92.41 (10.69)	91.97 (10.98)	0.120
Waist‐to‐hip ratio	0.88 (0.08)	0.88 (0.09)	0.87 (0.08)	<0.001
SBP (mmHg)	123.97 (17.72)	123.89 (17.45)	124.07 (18.01)	0.700
DBP (mmHg)	78.32 (10.66)	78.34 (10.54)	78.31 (10.80)	0.800
Fasting blood glucose (mmol/L)	4.77 (1.09)	4.78 (0.90)	4.77 (1.27)	0.003
Serum TC (mmol/L)	5.42 (1.07)	5.48 (1.05)	5.35 (1.10)	<0.001
Serum TG (mmol/L)	1.58 (1.31)	1.62 (1.28)	1.53 (1.35)	0.006
Serum LDL (mmol/L)	3.62 (0.91)	3.65 (0.90)	3.58 (0.91)	0.035
Serum HDL (mmol/L)	1.37 (0.35)	1.36 (0.35)	1.38 (0.35)	0.041
Uric acid (μmol/L)	279.03 (114.84)	284.81 (122.64)	272.66 (105.25)	<0.001
Total energy intake (kcal/d)	1856.40 (617.20)	1861.22 (607.68)	1851.06 (627.78)	0.400
Carbohydrate (g/d)	223.28 (41.18)	222.74 (39.07)	223.88 (43.40)	0.600
Dietary fiber intake (g/d)	10.69 (5.11)	10.60 (4.52)	10.80 (5.70)	0.500
Vegetables (g/d)	398.48 (208.45)	399.15 (183.77)	397.74 (232.87)	0.110
Fruit (g/d)	158.69 (216.35)	160.05 (218.82)	157.19 (213.65)	0.500
Fish (g/d)	59.95 (101.52)	57.48 (89.10)	62.68 (113.70)	0.500
Red meats (g/d)	87.42 (72.52)	88.91 (66.76)	85.76 (78.41)	0.034
Dairy intake (g/d)	137.15 (279.82)	137.94 (303.52)	136.29 (251.03)	0.200
Smoking, *n* (%)	395 (15%)	200 (14%)	195 (15%)	0.400
Alcohol consumption, *n* (%)	158 (5.9%)	81 (5.8%)	77 (6.0%)	0.800
Tea consumption, *n* (%)	1345 (50%)	719 (51%)	626 (49%)	0.300
Household income, *n* (%)				
≤1000	72 (2.7%)	29 (2.1%)	43 (3.4%)	0.060
1000–2000	860 (32%)	434 (31%)	426 (34%)	
2000–3000	1210 (46%)	657 (47%)	553 (44%)	
>3000	513 (19%)	273 (20%)	240 (19%)	
Education level, *n* (%)				
Middle school or lower	813 (30%)	389 (28%)	424 (33%)	0.005
High school or professional college	1244 (46%)	665 (47%)	579 (45%)	
University and upper	628 (23%)	352 (25%)	276 (22%)	
Calcium supplement, *n* (%)	862 (32%)	444 (32%)	418 (33%)	0.500
Multivitamins supplement, *n* (%)	581 (22%)	312 (22%)	269 (21%)	0.500

*Note*: Data are mean (SD) for continuous measures and *n* (%) for categorical measures. *p* value, significant statistic for two side.

Abbreviations: DBP, diastolic blood pressure; HDL‐C, high‐density lipoprotein cholesterol; LDL‐C, low‐density lipoprotein cholesterol; SBP, systolic blood pressure; TC, total cholesterol; TG, triglyceride.

### 2.3 Identification of protein biomarkers for osteoporosis

**FIGURE 2:**
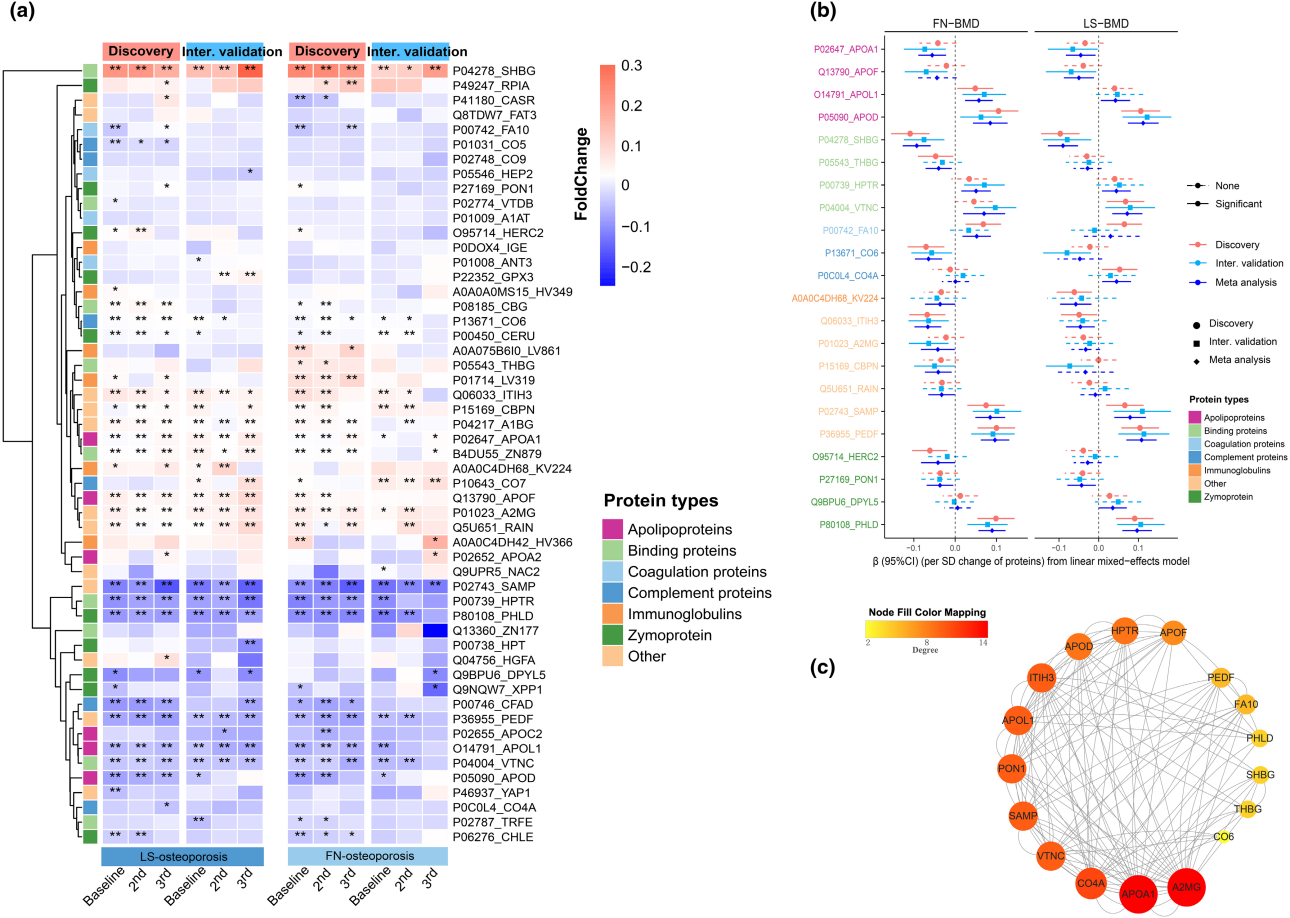
Prospective associations between serum proteins with osteoporosis and BMD. (a) The fold change of serum protein abundance between osteoporosis cases and controls at three time points. Fold change = (mean_‐OP_−mean_‐controls_)/mean_‐controls_. The Benjamini‐Hochberg (BH) false discovery rates (FDR) approach was applied to control alpha error. ***FDR <0.001, **FDR <0.01, *FDR <0.05. (b) The prospective associations between serum proteins and BMDs. The regression coefficients and 95% CIs (in SD/SD) between serum proteins and BMDs at the lumbar spine (LS) and femoral neck (FN) (*n* = 3244) were estimated by LMM model in the discovery and internal validation cohorts. The longitudinal BMD levels at three follow‐ups as dependent variables. The multivariate regressions were adjusted for baseline age, sex, BMI, waist‐hip ratio, educational level, household income, smoking status, alcohol drinking status, tea consumption, physical activity, total energy intake, total carbohydrate intake, dietary fiber intake, calcium supplement, multivitamins supplement, SBP, DBP, fasting blood glucose, TC, TG, LDL, HDL, and uric acid. The combined effects of proteins on BMDs from the discovery and internal validation cohorts were assessed using the meta‐analysis. The heterogeneity was investigated using the Cochran's Q and I‐square statistics. The Benjamini‐Hochberg (BH) false discovery rates (FDR) approach was applied to control alpha error. (c) The protein–protein interaction of serum proteins. BMD, bone mineral density; BMI, body mass index; CIs, confidence intervals; DBP, diastolic blood pressure; FN, femoral neck; HDL‐C, high‐density lipoprotein cholesterol; LDL‐C, low‐density lipoprotein cholesterol; LMM, linear mixed‐effects model; LS, lumbar spine; SBP, systolic blood pressure; SD, standard deviation; TC, total cholesterol; TG, triglyceride.

The longitudinal proteomics were profiled at three time points (Figure S2a). During the 9.8 follow‐up years, 2966 participants were tracked, with 1746 (LS‐OP = 499, FN‐OP = 330) in the discovery cohort and 1220 (LS‐OP = 337, FN‐OP = 191) in the internal validation cohort (Figure S2b). We identified 53 proteins associated with osteoporosis using LightGBM, of which 38 proteins were associated with LS‐OP (Figure S3a) and 28 proteins were associated with FN‐OP (Figure S3b). The Lasso regression uncovered 62 proteins associated with osteoporosis, of which 43 proteins were associated with LS‐OP and 32 proteins were associated with FN‐OP (Figure S4a). In the discovery cohort, the selected proteins accurately predicted the risk of LS‐OP (LightGBM‐_AUC_ = 0.76, Lasso‐_AUC_ = 0.74) and FN‐OP (LightGBM‐_AUC_ = 0.74, Lasso‐_AUC_ = 0.75) (Figure S3c,d, Figure S4b,c). The fold change in the discovery and internal validation cohorts allowed us to see the longitudinal differences in proteins between the OP group and controls (Figure 2a). The meta‐analysis revealed that 22 of these proteins had a significant association with BMD (*p* < 0.05, heterogeneity‐*I* ^*2*^ < 30%) (Table S4, Figure 2b). The proteins of A2MG, APOA1, CO4A, VTNC, and SAMP were the top five most dominant proteins in protein–protein interaction network (Figure 2c).

Additionally, the LCTM model revealed three latent trajectories of BMD (sustained low, medium, and high) at LS and FN anatomical sites (Figure S5a,b). The serum proteins were significantly dysregulated among different trajectories. They were mostly composed of the proteins PHLD, SAMP, PEDF, HPTR, APOA1, SHBG, CO6, A2MG, CBPN, RAIN APOD, and THBG (Figure S6a,b).

### 2.4 Association between PRS and BMD/osteoporosis risk

**FIGURE 3:**
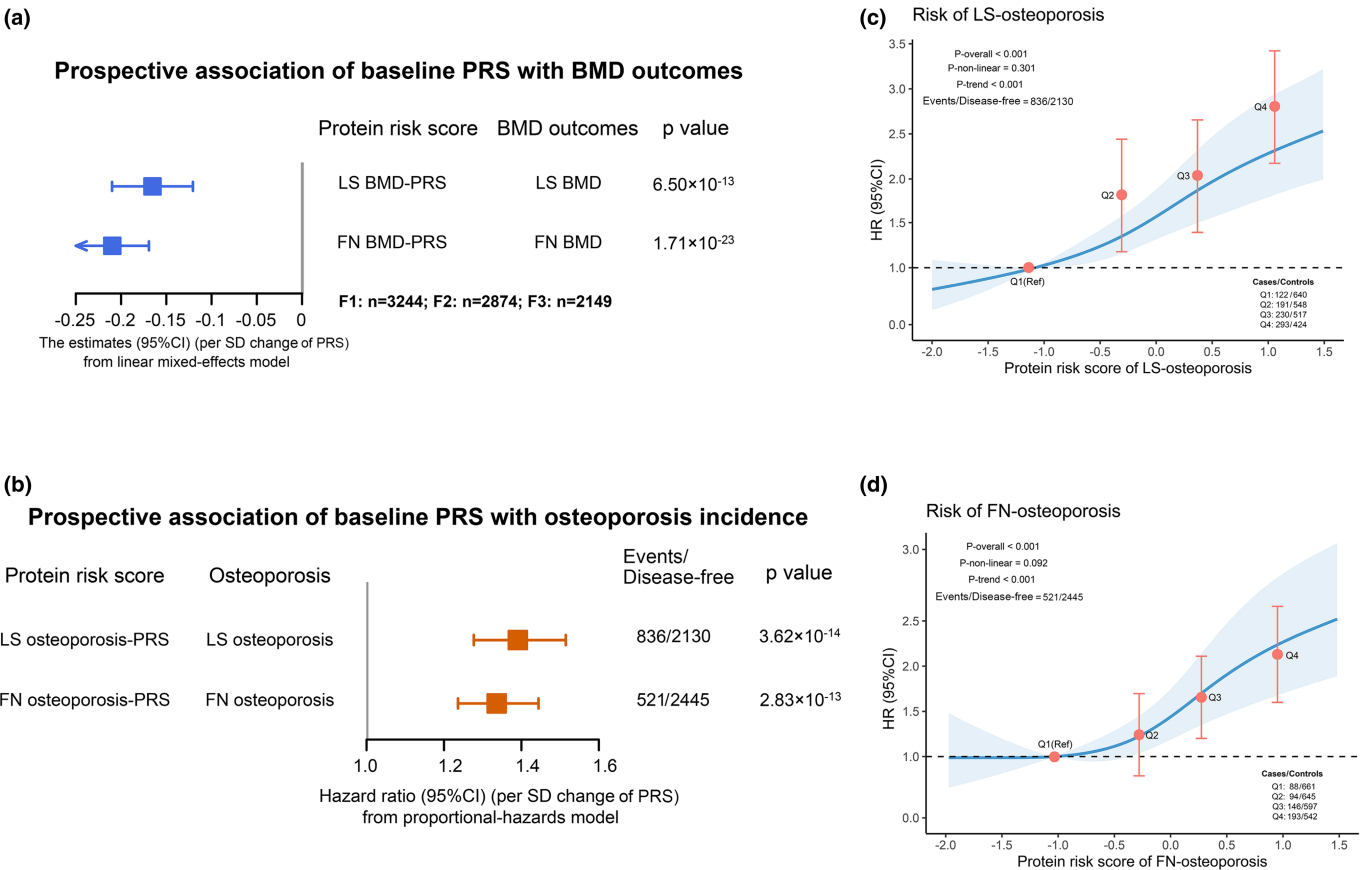
Prospective associations of PRS with osteoporosis and BMD. (a) Regression coefficients and 95% CIs (in SD/SD) between baseline protein risk score (PRS) and BMDs. The LMM was used to analyze the associations between baseline PRS and BMD at the LS and FN. (b) The associations between baseline PRS and osteoporosis risk at the LS and FN analyzed using Cox regression. (c,d) Dose–response associations between the baseline PRS and osteoporosis risk at the LS and FN were analyzed by the restricted cubic spline model. The corresponding hazard ratios (95% CIs) for the quartiles (Q1–Q4) were estimated using the Cox regression model. All the analyses were adjusted for baseline age, sex, BMI, waist‐hip ratio, educational level, household income, smoking status, alcohol drinking status, tea consumption, physical activity, total energy intake, total carbohydrate intake, dietary fiber intake, calcium supplement, multivitamins supplement, SBP, DBP, fasting blood glucose, TC, TG, LDL, HDL, and uric acid. BMD, bone mineral density; BMI, body mass index; CIs, confidence intervals; DBP, diastolic blood pressure; FN, femoral neck; HDL‐C, high‐density lipoprotein cholesterol; LDL‐C, low‐density lipoprotein cholesterol; LMM, linear mixed‐effects model; LS, lumbar spine; SBP, systolic blood pressure; SD, standard deviation; TC, total cholesterol; TG, triglyceride.

**FIGURE 4:**
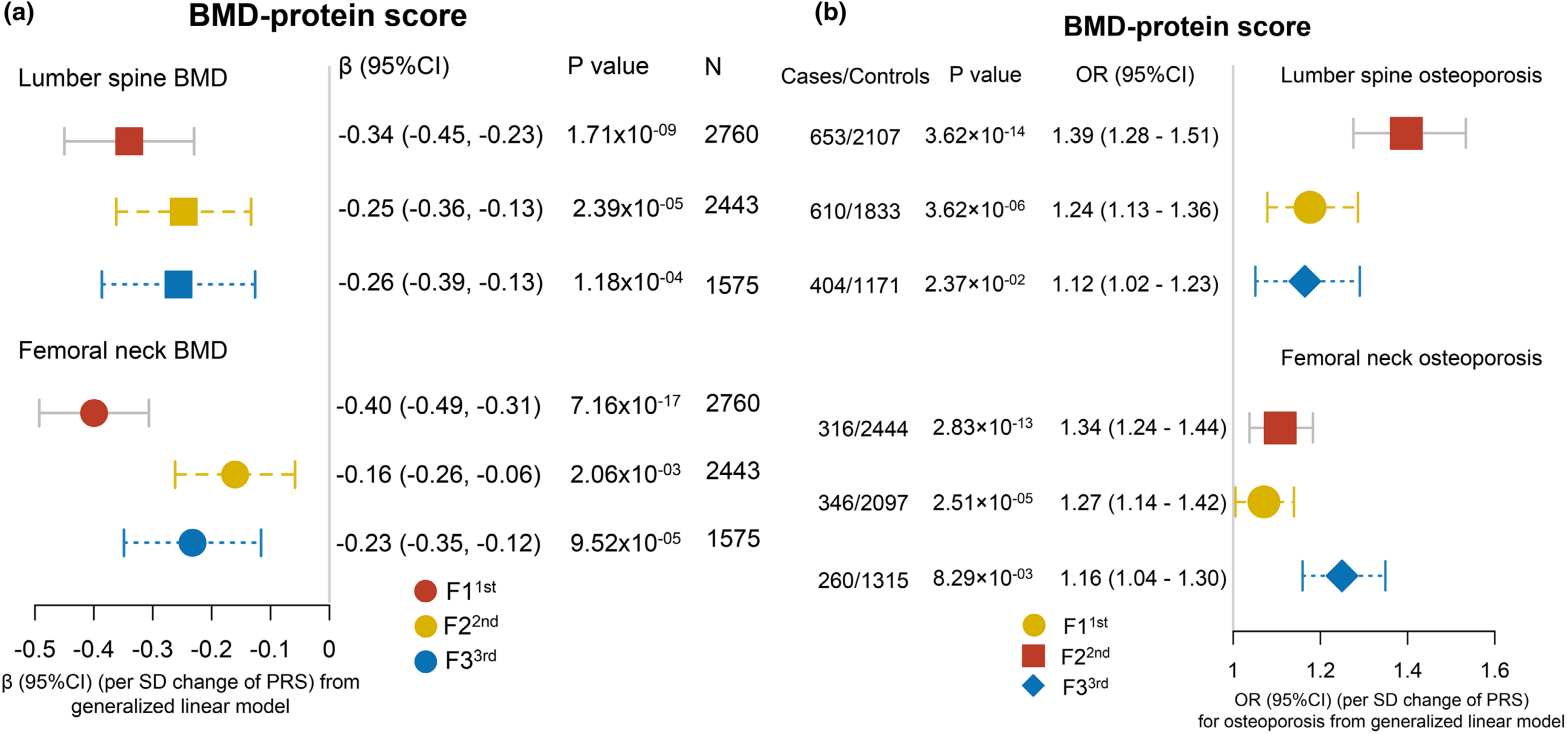
The cross‐sectional associations of PRS with osteoporosis risk and BMD at three follow‐up visits. The cross‐sectional associations of protein risk score (PRS) with BMD (a) and osteoporosis risk (b) at each visit. The PRS was constructed using the protein concentrations at the baseline, second, and third follow‐ups as well as the beta coefficients from the meta‐analysis. The cross‐sectional associations were replicated using the PRS and BMD/osteoporosis data that determined at the baseline, 2nd, and 3rd follow‐ups and analyzed by generalized linear models (GLM). Covariates adjusted: see Figure 3.

We assembled the bone site‐specific protein risk scores (PRS) to examine the joint “effects” of identified protein biomarkers on BMD and osteoporosis risk by repeating the analyses as done above for the individual proteins. The LMM showed that the PRS was inversely associated with LS‐BMD (*β* = −0.17, 95% CI: −0.21, −0.12, *p* = 6.50 × 10^−13^) and FN‐BMD (*β* = −0.21, 95% CI: −0.25, −0.17, *p* = 1.71 × 10^−23^) in the longitudinal study about 9.8 follow‐up years (Figure 3a). Additionally, we separately explored cross‐sectional associations and discovered similar results between PRS and BMD using protein biomarkers and BMD data derived at three time periods (LS‐BMD: *β* = −0.26, 95% CI: −0.39, −0.13, *p* = 1.18 × 10^−04^; FN‐BMD: *β* = −0.23, 95% CI: −0.35, −0.12, *p* = 9.52 × 10^−05^) (Figure 4a). This was done to confirm the constancy of the PRS‐BMD associations over time.

The Cox proportional hazard models showed that HRs (95% CIs) of osteoporosis for each SD increase in baseline PRS were HR: 1.39 (95% CI: 1.28–1.51), *p* = 3.62 × 10^−14^ at LS, and HR: 1.34 (95% CI: 1.24–1.44), *p* = 2.83 × 10^−13^ at FN (Figure 3b). We also explored the dose–response associations between baseline PRS with LS‐osteoporosis (Figure 3c) and FN‐osteoporosis (Figure 3d) risk by the restricted cubic spline. The hazards ratio (95% CI) of osteoporosis for the highest (vs. lowest) quartile of PRS were 2.74 (95% CI: 3.42–2.06) and 2.12 (95% CI: 2.63–1.61) at the LS and FN (*p*‐trend <0.001) (Figure 3c,d). The consistency analyses showed the negative associations between PRS and osteoporosis risk at the three visits in cross‐sectional analyses (LS‐OP: OR = 1.12, 95% CI: 1.02, 1.23, *p* = 2.37 × 10^−02^; FN‐OP: OR = 1.16, 95% CI: 1.04, 1.30, *p* = 8.29 × 10^−03^) (Figure 4b).

### 2.5 Mendelian randomization analyses between plasma proteins and BMD

In this section, we conducted two‐sample Mendelian randomization (MR) analyses to verify the potential causal relationship between the identified protein biomarkers with osteoporosis and BMD in East Asian and European populations using public GWAS‐summary data. Totally, 32 SNPs were selected as genetic instruments for 13 serum proteins from East Asian population (Table S5), and 33 SNPs were selected as genetic instruments for 22 plasma proteins from European population (Tables S6,S7). The public GWAS‐summary data of proteins, osteoporosis, and BMDs were presented in Tables S5–S7. We found that serum paraoxonase/arylesterase 1 (PON1) was positively associated with osteoporosis in East Asian (Figure S7a, Table S8), while cholinesterase (BCHE) was significantly associated with LS‐BMD, and apolipoprotein L1 (APOL1) was significantly associated with FN‐BMD and heel eBMD in European population (Figure S7b–d, Table S9).

### 2.6 Biological ages and OP

**FIGURE 5:**
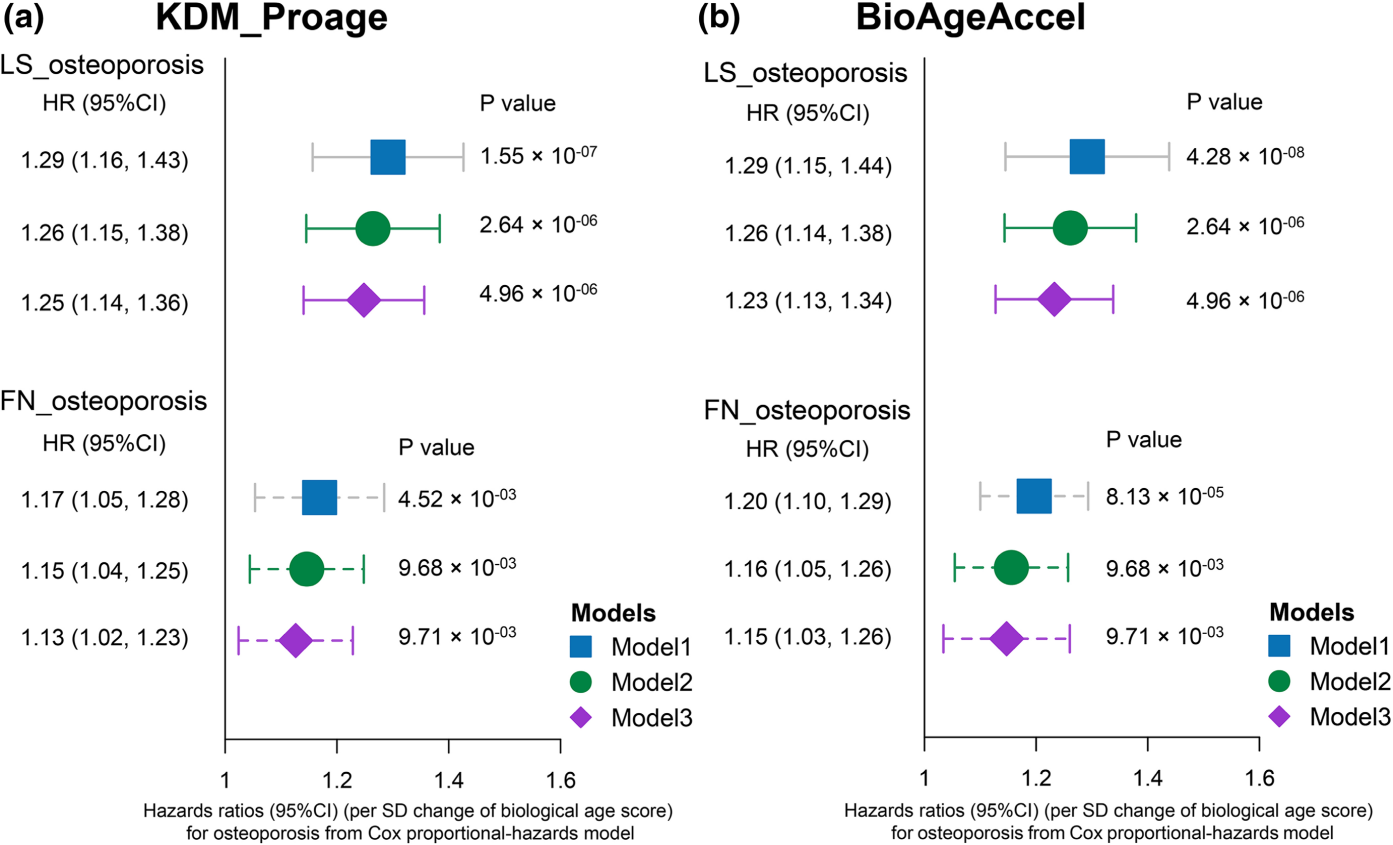
The prospective associations between biological age scores and osteoporosis risk. The prospective associations of KDM‐Proage (a) and BioAgeAccel (b) with osteoporosis risk. The associations between baseline biological age scores (in per SD change) and osteoporosis risk were analyzed using Cox regression. Model 1 was adjusted for baseline age and sex. Model 2 was adjusted for Model 1 + BMI, waist‐hip ratio, educational level, household income, smoking status, alcohol drinking status, tea consumption, physical activity, total energy intake, total carbohydrate intake, dietary fiber intake, calcium supplement, and multivitamins supplement. Model 3 was adjusted for Model 2 + SBP, DBP, fasting blood glucose, TC, TG, LDL, HDL, and uric acid. BMD, bone mineral density; BMI, body mass index; CIs, confidence intervals; DBP, diastolic blood pressure; FN, femoral neck; HDL‐C, high‐density lipoprotein cholesterol; LDL‐C, low‐density lipoprotein cholesterol; LMM, linear mixed‐effects model; LS, lumbar spine; SBP, systolic blood pressure; SD, standard deviation; TC, total cholesterol; TG, triglyceride.

After removed the redundant features, the chronological age (CA) showed a strong relationship with 12 proteins (Figure S8a). Finally, Klemera and Doubal proteomics age (KDM‐Proage) and BioAgeAccel (biological age acceleration) were generated. The KDM‐Proage was significantly and positively correlated with chronological age (*r* = 0.16, *p* < 2.2 × 10^−16^) (Figure S8b). Meanwhile, the hazard ratios of osteoporosis for each SD increase in KDM‐Proage were (HR: 1.25, 95% CI: 1.14–1.36, *p* = 4.96 × 10^−06^) and (HR: 1.13, 95% CI: 1.02–1.23, *p* = 9.71 × 10^−03^) at LS and FN (Figure 5a), and in BioAgeAcce were (HR: 1.23, 95% CI: 1.13–1.34, *p* = 4.96 × 10^−06^) and (HR: 1.15, 95% CI: 1.03–1.26, *p* = 9.71 × 10^−03^) at LS and FN (Figure 5b).

## 3 DISCUSSION

In this study, we identified 53 osteoporosis‐associated proteins, of which 22 exhibited significant correlations with bone mineral density (BMD). The protein risk score that was constructed by identified proteins exerted joint effects on osteoporosis and bone loss. The noteworthy proteins included PHLD, SAMP, PEDF, HPTR, APOA1, SHBG, CO6, A2MG, CBPN, RAIN APOD, and THBG. These serum proteins can be served as indicators for assessing bone aging, also known as biological age. Apolipoproteins, zymoproteins, complements, and binding proteins have emerged as possible biomarkers to shed light on the molecular mechanisms of osteoporosis.

### 3.1 Apolipoproteins and osteoporosis

Apolipoproteins may be risk factors for atherosclerosis and cardiovascular disease, according to earlier research (Frikke‐Schmidt, 2020; Mehta & Shapiro, 2022). A considerable link between apolipoproteins and the development of osteoporosis, however, has frequently been observed in recent years (Martineau et al., 2016; Wang et al., 2023). In this study, APOA1 and APOF were found to be significantly negatively associated with BMD, whereas APOL1 and APOD were significantly positively associated with BMD.

In addition to the well‐recognized antiatherogenic effects, emerging evidence highlighted that HDL‐c and its major protein component of apolipoprotein A1 (APOA1) also play more functional roles in other biological processes, including systemic inflammation, nitric oxide production, oxidative stress, and regulation of bone metabolism homeostasis (Ouweneel et al., 2022). The cross‐sectional study showed that APOA1 positively associated with osteocalcin (OC), L1‐L4 BMD, and T‐score in Chinese postmenopausal women. Additionally, it has been demonstrated that APOA1 is independently linked to a decreased risk of osteoporosis (Wang et al., 2023). The results showed that the risk of osteoporosis significantly decreased with the increase of APOA1. Apolipoprotein D (ApoD) is a member of the lipocalin family known to transport small hydrophobic ligands that occurs in the macromolecular complex with lecithin‐cholesterol acyltransferase, which was relate to the extracellular matrix and cell adhesion processes in osteoarthritis synovium, and associated with molecular heterogeneity in low‐grade knee osteoarthritis cartilage (Steinberg et al., 2021). In additional, the results indicate that ApoD is upregulated in osteoblasts under conditions that reduce proliferation rate. In accordance with its suggested role in osteoblast function, ApoD‐null female mice display lower trabecular and cortical bone volumes with concurrent higher osteoblast surface and number of osteoclasts. Only cortical bone volume was reduced in ApoD‐null male mice, arguing for gender‐specific interactions (Martineau et al., 2016).

### 3.2 Cardiometabolic and aging‐related proteins and bone health

Sex hormone binding proteins (SHBG) are carriers of sex hormones, which are significantly associated with cardiovascular risk (Yeap et al., 2022), fatty liver (Wang, Xie, et al., 2020), hip fracture (Rosenberg et al., 2021), and aging‐related changes in bone mass and size (Banica et al., 2022). The SHBG protein have been previously associated with fracture risk by serum proteomics analysis (Nielson et al., 2017). Thyroxine‐binding globulin (THBG) is a major transporter protein for thyroid hormones. The skeleton is an exquisitely sensitive and archetypal T3‐target tissue that demonstrates the critical role for thyroid hormones during development, linear growth, and adult bone turnover and maintenance (Lademann et al., 2020). Thyrotoxicosis is an established cause of secondary osteoporosis, and abnormal thyroid hormone signaling has recently been identified as a novel risk factor for osteoarthritis (Li et al., 2019). The bioactive core vitronectin (VTNC)‐derived peptide (VnP‐16) promoted bone formation by accelerating osteoblast differentiation and activity through direct interaction with *β*1 integrin followed by FAK activation. VnP‐16 had a strong anabolic effect on bone regeneration by stimulating osteoblast differentiation and increasing osteoblast number, and significantly alleviated proinflammatory cytokine‐induced bone resorption by restraining osteoclast differentiation and function in murine models (Min et al., 2018). A vitronectin‐derived peptide prevents and restores alveolar bone loss by modulating bone remodeling and expression of RANKL and IL‐17A (Lee et al., 2022).

### 3.3 Immunity and inflammation‐related proteins and BMD

This study showed that the immunoproteins and complement protein were significantly associated with BMD, such as immunoglobulin kappa variable 2–24 (KV224), complement 6 (CO6), and complement C4A (C4‐A). Previous studies suggested that the immune system is highly linked to the skeletal system and actively involved in the pathophysiology of osteoporosis (Westhrin et al., 2020). The innate immune cells modulate osteoporosis by producing several proinflammatory mediators (such as interleukin‐1‐beta, IL‐1*β*; interleukine‐6, IL‐6; tumor necrosis factor‐α, TNF‐α, and toll‐like receptor 4, TLR4) and affecting the receptor activator of nuclear factor‐κB (RANK)/ligand for a RANK receptor (RANKL)/osteoprotegerin (OPG) axis (Breedveld et al., 2021). The B lymphocytes are a major regulator for forming osteoclasts by granulocyte colony‐stimulating factor secretion and the RANKL/osteoprotegerin system (Choi et al., 2021). Using a novel high‐throughput serum proteomics study in a population‐based cohort of older men, the BMD‐loss proteins were identified and were enriched in biological processes related to innate immune response and inflammation, and several additional proteins are related to biological processes relevant to bone (Nielson et al., 2017).

### 3.4 Advantages and limitations of study

Many advantages of this study should be mentioned. First, our study is the first to examine the associations of serum proteins with BMD and the risk of osteoporosis using the proteome‐wide technique in a 9.8‐year longitudinal cohort. Second, the repeated measures of serum proteomics and BMD demonstrated the consistency of the protein‐BMD association at three time point. The longitudinal trajectory analysis of BMD uncovered novel biomarkers for longitudinal change in BMD. Third, we generated the biological ages (BAs) with BMD‐proteins by the Klemera and Doubal method (KDM) to investigate the bone aging.

Some limitations in this study need to be noticed. First, we cannot determine the causal relationships between all of the serum proteins and osteoporosis due to the inherent limitations of observational study. The underlying mechanisms of proteome in osteoporosis needed to be confirmed in animal model. Second, the serum proteome can vary significantly among individuals due to factors like age, sex, genetics, and comorbidities. This heterogeneity can complicate data interpretation. Third, the limited summary data from GWAS studies of plasma proteomics leaded to the causal relationship could not be verified for all identified proteins through MR analysis.

## 4 CONCLUSIONS

Our research showed that apolipoproteins, zymoproteins, complements, and binding proteins have become the promising targets for therapeutic interventions of osteoporosis. These serum proteins can be used to assess the biological age of bone and the rate at which the bone is aging.

## 5 MATERIALS AND METHODS

### 5.1 Study participants

The study was based on the community‐based prospective cohort Guangzhou Nutrition and Health Study (GNHS, ClinicalTrials.gov identifier: NCT03179657) that consisted of 4048 apparently healthy participants aged 45–75 years at baseline between 2008 and 2013 (Ling et al., 2023). The metadata from questionnaire interviews, anthropometric measurements, and blood samples were collected every 3 years between 2008 and 2019 (Long et al., 2021). The population cohort was divided into the discovery (*n* = 1785) and internal validation (*n* = 1630) cohorts based on test batches (1st and 2nd) of serum proteome at up to three time points: baseline (between 2008 and 2013), the second follow‐up (between 2014 and 2017), and third follow‐up (between 2018 and 2019). The exclusion criteria for participants at baseline were as follows: (1) Missing important variables (age, sex, and so on) at baseline and missing BMD measures at 1st follow‐up: 206; (2) Patients with history of fracture and disease that affects BMD at baseline (hyperthyroidism, cancer, and uremia): 337; (3) Without proteomics data at baseline: 261. Finally, 3244 participants at baseline were retained for further study (Figure S9).

The study protocol was approved by the Ethics Committee of the School of Public Health at Sun Yat‐sen University. All participants provided written informed consent before the investigation.

### 5.2 BMD measurements and osteoporosis definition

The data collection about detailed demographic characteristics were included in Appendix S1. BMD (g/cm^2^) at the lumbar spine (L1‐L4, LS) and femoral neck (FN) were measured from the first to the third follow‐up visits by a dual‐energy X‐ray absorptiometry (DXA) (Discovery W; Hologic Inc., Waltham, MA, USA). The in vivo variation coefficients of measurements in all participants after repositioning were 0.94% (LS) and 0.71% (FN). Osteoporosis was defined as a T‐score of less than −2.5 and osteopenia was between −2.5 and −1 (Kanis et al., 2008; Looker et al., 2012) or currently under medical treatment for osteoporosis at any follow‐up visits.

### 5.3 Serum proteome analysis

Briefly, serum proteome analysis was performed as previously described (Cai et al., 2023). The peptides were extracted from the serum samples and were then digested with a two‐step overnight tryptic digestion (Hualishi Tech. Ltd, Beijing, China) at 32°C for 4 h and 12 h, using an enzyme‐to‐substrate ratio of 1:60 (final ratio 1:30) for each digestion step. The SWATH‐MS analysis for the peptide samples were perform on an Eksigent NanoLC 400 System (Eksigent, Dublin, CA, USA) coupled with a TripleTOF 5600 system (SCIEX, CA, USA). The MS files were analyzed using DIA‐NN (1.8) against a plasma spectral library containing 5102 peptides and 819 unique proteins from the Swiss‐Prot database of *Homo sapiens*. Protein inference was set to the protein names (from the FASTA file), and the cross‐run normalization was set as “RT dependent”. A total of 413 proteins from ~20,000 proteomes were quantified. The detailed methods of LC–MS/MS were provided in Appendix S1.

### 5.4 Statistical analysis

#### 5.4.1 Characteristics of the participants

Chi‐square tests and *t* tests were used to assess participants' demographic characteristics by the discovery and internal validation cohorts and OP status in different cohorts. The continuous variables were presented as means and standard deviations (SD), and categorical variables as counts and percentages.

#### 5.4.2 Latent class trajectory analysis

We employed the latent class trajectory model (LCTM) (Mirza et al., 2016) to explore the longitudinal trajectories of BMD outcomes at two anatomical locations across three follow‐up visits within 6.6 years. We fitted models with one to five groups and confirmed the optimal model based on Bayesian information criterion (BIC) and average posterior probabilities of assignment. The change of BMD across 1st, 2nd, and 3rd study visits were evaluated by paired *t* test.

#### 5.4.3 Machine learning frameworks for data integration and explanation

The missing value of serum protein was filled with 1/2 of the lowest value in all analyzed samples. The abundances of proteins were normalized to an average of 0 and a standard deviation (SD) of 1. The difference of proteomic matrixes between the discovery and internal validation cohorts at baseline were evaluated by principal component analysis (PCA).

We employed a model based on a gradient boosting framework‐Light Gradient Boosting Machine (LightGBM) to identify the proteomic biomarkers for OP by 2‐step analysis. (Gou et al., 2021). Firstly, we divided the discovery cohort into training set (*n* = 1222) and testing set (*n* = 524) using a random number generator by the set‐aside method. Then, proteomic features were selected from the discovery cohort using the Shapley Additive exPlanations (SHAP). The mean absolute value of the SHAP values for each feature represents their average contribution to the overall model predictions. Thus, features with an average absolute SHAP value over 0 were used as selected features. Secondly, the preselected features were further confirmed in the internal validation cohort. We then tested the model with the internal testing dataset and the independent internal validation cohort.

The LASSO (Least Absolute Shrinkage and Selection Operator) regression was performed using a ten‐fold cross validation in the discovery cohort, and the model was validated in an internal validation cohort (Bose et al., 2022).

#### 5.4.4 Longitudinal proteome‐wide analysis with BMD/osteoporosis

The data of proteomics and BMD in all analyses were standardized to an average of 0 and a standard deviation (SD) of 1. The associations between baseline serum proteins and BMDs at the lumbar spine (LS) and femoral neck (FN) were estimated, respectively, by linear mixed‐effects model (LMM) in the discovery and internal validation cohorts. The dependent variables were the BMD levels at three follow‐ups. All the analyses were adjusted for baseline age, sex, BMI, waist‐hip ratio, educational level, household income, smoking status, alcohol drinking status, tea consumption, physical activity, total energy intake, total carbohydrate intake, dietary fiber intake, calcium supplement, multivitamins supplement, SBP, DBP, fasting blood glucose, TC, TG, LDL, HDL, and uric acid. The combined effects of proteins on BMDs from the discovery and internal validation cohorts were assessed using the random‐effects meta‐analysis. The heterogeneity was investigated using the Cochran's Q and I‐square statistics. The Benjamini‐Hochberg (BH) false discovery rates (FDR) approach was applied to control alpha error. The linear mixed‐effects model contains a random intercept on the participants identifier to adjust for the heterogeneity of dependent variables. We used one‐way ANOVA to evaluate the different expression of protein biomarkers among the different longitudinal trajectories of BMD.

#### 5.4.5 Protein risk score analysis with BMD/osteoporosis

We constructed a protein risk score (PRS) (Appendix S1) based on the identified BMD‐specific protein biomarkers from meta‐analysis to evaluate the joint association of proteins with BMD and osteoporosis risk in all participants. We combined the internal validation and discovery cohorts into a total population in this study for later analysis. The prospective association between baseline PRS with BMDs and osteoporosis risk from three follow‐ups were examined by LMM and Cox regression, respectively. Moreover, the baseline PRS was handled as quarters (categorical) and continuous to estimate the on‐linear associations and trend by the restricted cubic spline (RCS) regression. Meanwhile, we also employed the generalized linear model (GLM) to uncover the cross‐sectional association of PRS with BMD and osteoporosis risk at the corresponding time point. All study adjusted for the same covariates as described above.

#### 5.4.6 Mendelian randomisation analysis

The two‐sample MR analysis with public GWAS‐summary data from East Asian and European was conducted to validated the causality between proteins and bone health (Chen et al., 2019). The selection criteria of genetic instruments for proteins were included in Appendix S1. The putatively causal variants (*p* value ≤5 × 10^−8^) of circulating proteins were extracted from Chinese (Xu et al., 2023) and European populations (Emilsson et al., 2018; Suhre et al., 2017; Sun et al., 2018; Yao et al., 2018). The GWAS‐summary data of osteoporosis were extracted from Japanese (Ishigaki et al., 2020). The SNP‐BMD coefficients for LS and FN were extracted from the GWAS study in European populations (Zheng et al., 2015). The GWAS‐summary data of eBMD were estimated by heel quantitative ultrasound in UK biobank (Al‐Ansari et al., 2022). The main assumptions of two‐sample MR analysis were shown in Figure S10. The MR Egger, Weighted median, inverse variance weighted (IVW), simple mode, and weighted mode were performed for multiple genetic SNP‐instruments, and Wald ratio was performed for single SNP‐instrument using the “TwoSampleMR” package.

#### 5.4.7 Biological ages constructions

The Klemera and Doudal algorithm (Klemera & Doubal, 2006) was used for the construction of biological ages (Appendix S1). Briefly, the KD method was consisted of two steps to convert proteomic features into aging rate and making them comparable. The first step is regressing every protein to chronological age. By doing this, we gained the estimated age as well as its standard error using a particular biomarker. Every protein was processed and all the estimated ages have the unit “year” which is the same as chronological age. We consider the regression step as a normalization process that make different markers comparable in terms of unit. The second step is aggregating the age estimates of each protein as well as chronological age and construct biological ages (Nie et al., 2022).

We used python 3.11.5 to perform a LightGBM analysis, Lasso regression, and model explanation, R [R Foundation for Statistical Computing (software version 4.2.1)] for statistical analysis unless otherwise specified, and *p* value <0.05 was considered statistically significant and all *p* values were 2‐sided. The Protein–Protein Interaction (PPI) network from STRING was visualized by Cytoscape software.

## AUTHOR CONTRIBUTIONS

Y.‐m.C., J.‐S.Z., and T.G. contributed to study conceptualization and design. J.X., X.C., and Z.M. contributed to the data analysis. J.X., X.C., Z.M., Y.Y., D.C. Z.X.Y., L.Y., W.H., L.Z., J.T.W., Z.X., Y.F., and Y.X. contributed to data collection. J.‐J.X. contributed to writing the manuscript. Y.‐m.C., J.‐S.Z., and T.G. contributed to writing, reviewing, and editing the manuscript. All authors read, revised, and approved the final draft. Y.‐m.C. and J.‐S.Z. are the guarantors of this work and, as such, had full access to all the data in the study and take responsibility for the integrity of the data and the accuracy of the data analysis.

## FUNDING INFORMATION

This study was jointly supported by the National Natural Science Foundation of China (No. 82073546, 82073529, and 81773416); the Shenzhen Fundamental Research Program (JCYJ20210324125202006); and the 5010 Program for Clinical Researches (No. 2007032) of the Sun Yat‐sen University (Guangzhou, China). The funding sponsors had no role in the design of the study; in the collection, analyses, or interpretation of data; in the writing of the manuscript, and in the decision to publish the results.

## CONFLICT OF INTEREST STATEMENT

The authors declare that they have no competing interests.

## CONSENT

All participants gave written informed consent.

## Supporting information


Figures S1–S10

Tables S1–S9

Appendix S1

## Data Availability

The mass spectrometry proteomics data have been deposited to the ProteomeXchange Consortium (http://proteomecentral.proteomexchange.org) via the iProX partner repository with the dataset identifier PXD019675. All data supporting the conclusions of the article are presented in the main text and Appendix S1.
