# Supplementary material for: Proteome‐wide profiling reveals dysregulated molecular features and accelerated aging in osteoporosis: A 9.8‐year prospective study
Source: Aging Cell. 2023 Nov 16;23(2):e14035. doi: 10.1111/acel.14035 (PMC10861190; doi:10.1111/acel.14035)
Supplement: Supplementary file 1 — Figures S1–S10 [file ACEL-23-e14035-s002.zip › acel14035-sup-0003-FigureS3.pdf]

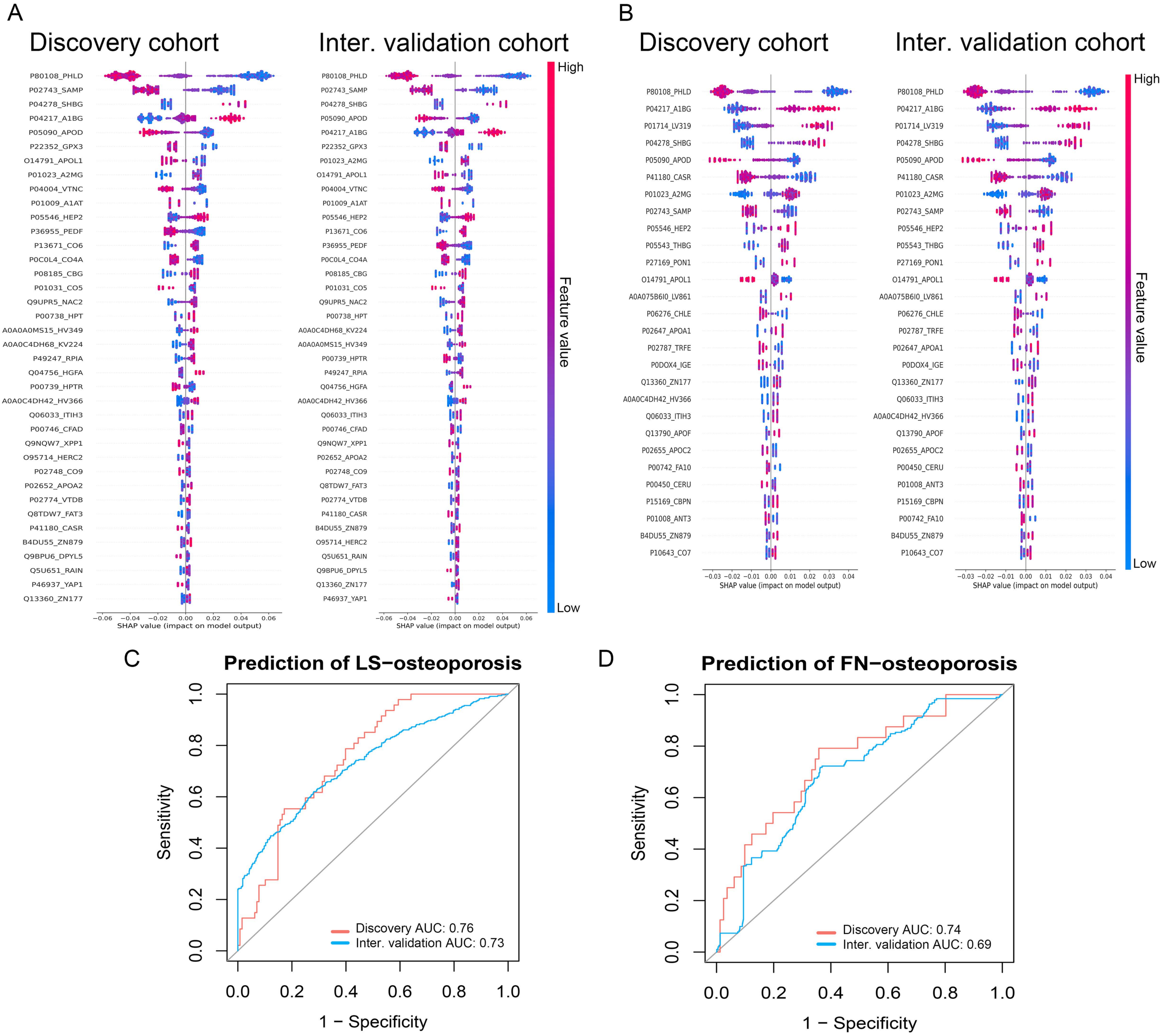
A
Discovery cohort
P80108_PHLD
P02743_SAMP
P04278_SHBG
P04217_A1BG
P05090_APOD
P22352_GPX3
O14791_APOL1
P01023_A2MG
P04004_VTNC
P01009_A1AT
P05546_HEP2
P36955_PEDF
P13671_CO6
P0C0L4_CO4A
P08185_CBG
P01031_CO5
Q9UPR5_NAC2
P00738_HPT
A0A0A0MS15_HV349
A0A0C4DH68_KV224
P49247_RPIA
Q04756_HGFA
P00739_HPTR
A0A0C4DH42_HV366
Q06033_ITIH3
P00746_CFAD
Q9NQW7_XPP1
O95714_HERC2
P02748_CO9
P02652_APOA2
P02774_VTDB
Q8TDW7_FAT3
P41180_CASR
B4DU55_ZN879
Q9BPU6_DPYL5
Q5U651_RAIN
P46937_YAP1
Q13360_ZN177
−0.06 −0.04 −0.02 0.00 0.02 0.04 0.06
SHAP value (impact on model output)
Inter. validation cohort
P80108_PHLD
P02743_SAMP
P04278_SHBG
P05090_APOD
P04217_A1BG
P22352_GPX3
P01023_A2MG
O14791_APOL1
P04004_VTNC
P01009_A1AT
P05546_HEP2
P13671_CO6
P36955_PEDF
P0C0L4_CO4A
P08185_CBG
P01031_CO5
Q9UPR5_NAC2
P00738_HPT
A0A0C4DH68_KV224
A0A0A0MS15_HV349
P00739_HPTR
P49247_RPIA
Q04756_HGFA
A0A0C4DH42_HV366
Q06033_ITIH3
P00746_CFAD
Q9NQW7_XPP1
P02652_APOA2
P02748_CO9
Q8TDW7_FAT3
P02774_VTDB
P41180_CASR
B4DU55_ZN879
O95714_HERC2
Q5U651_RAIN
Q9BPU6_DPYL5
Q13360_ZN177
P46937_YAP1
−0.06 −0.04 −0.02 0.00 0.02 0.04 0.06
SHAP value (impact on model output)
High
Feature value
Low
B
Discovery cohort
P80108_PHLD
P04217_A1BG
P01714_LV319
P04278_SHBG
P05090_APOD
P41180_CASR
P01023_A2MG
P02743_SAMP
P05546_HEP2
P05543_THBG
P27169_PON1
O14791_APOL1
A0A075B6I0_LV861
P06276_CHLE
P02647_APOA1
P02787_TRFE
P0DOX4_IGE
Q13360_ZN177
A0A0C4DH42_HV366
Q06033_ITIH3
Q13790_APOF
P02655_APOC2
P00742_FA10
P00450_CERU
P15169_CBPN
P01008_ANT3
B4DU55_ZN879
P10643_CO7
−0.03 −0.02 −0.01 0.00 0.01 0.02 0.03 0.04
SHAP value (impact on model output)
Inter. validation cohort
P80108_PHLD
P04217_A1BG
P01714_LV319
P04278_SHBG
P05090_APOD
P41180_CASR
P01023_A2MG
P02743_SAMP
P05546_HEP2
P05543_THBG
P27169_PON1
O14791_APOL1
A0A075B6I0_LV861
P06276_CHLE
P02787_TRFE
P02647_APOA1
P0DOX4_IGE
Q13360_ZN177
Q06033_ITIH3
A0A0C4DH42_HV366
Q13790_APOF
P02655_APOC2
P00450_CERU
P01008_ANT3
P15169_CBPN
P00742_FA10
B4DU55_ZN879
P10643_CO7
−0.03 −0.02 −0.01 0.00 0.01 0.02 0.03 0.04
SHAP value (impact on model output)
High
Feature value
Low
C
Prediction of LS−osteoporosis
Sensitivity
0.0 0.2 0.4 0.6 0.8 1.0
1 − Specificity
Discovery AUC: 0.76
Inter. validation AUC: 0.73
D
Prediction of FN−osteoporosis
Sensitivity
0.0 0.2 0.4 0.6 0.8 1.0
1 − Specificity
Discovery AUC: 0.74
Inter. validation AUC: 0.69
