# Supplementary material for: Proteome‐wide profiling reveals dysregulated molecular features and accelerated aging in osteoporosis: A 9.8‐year prospective study
Source: Aging Cell. 2023 Nov 16;23(2):e14035. doi: 10.1111/acel.14035 (PMC10861190; doi:10.1111/acel.14035)
Supplement: Supplementary file 1 — Figures S1–S10 [file ACEL-23-e14035-s002.zip › acel14035-sup-0004-FigureS4.pdf]

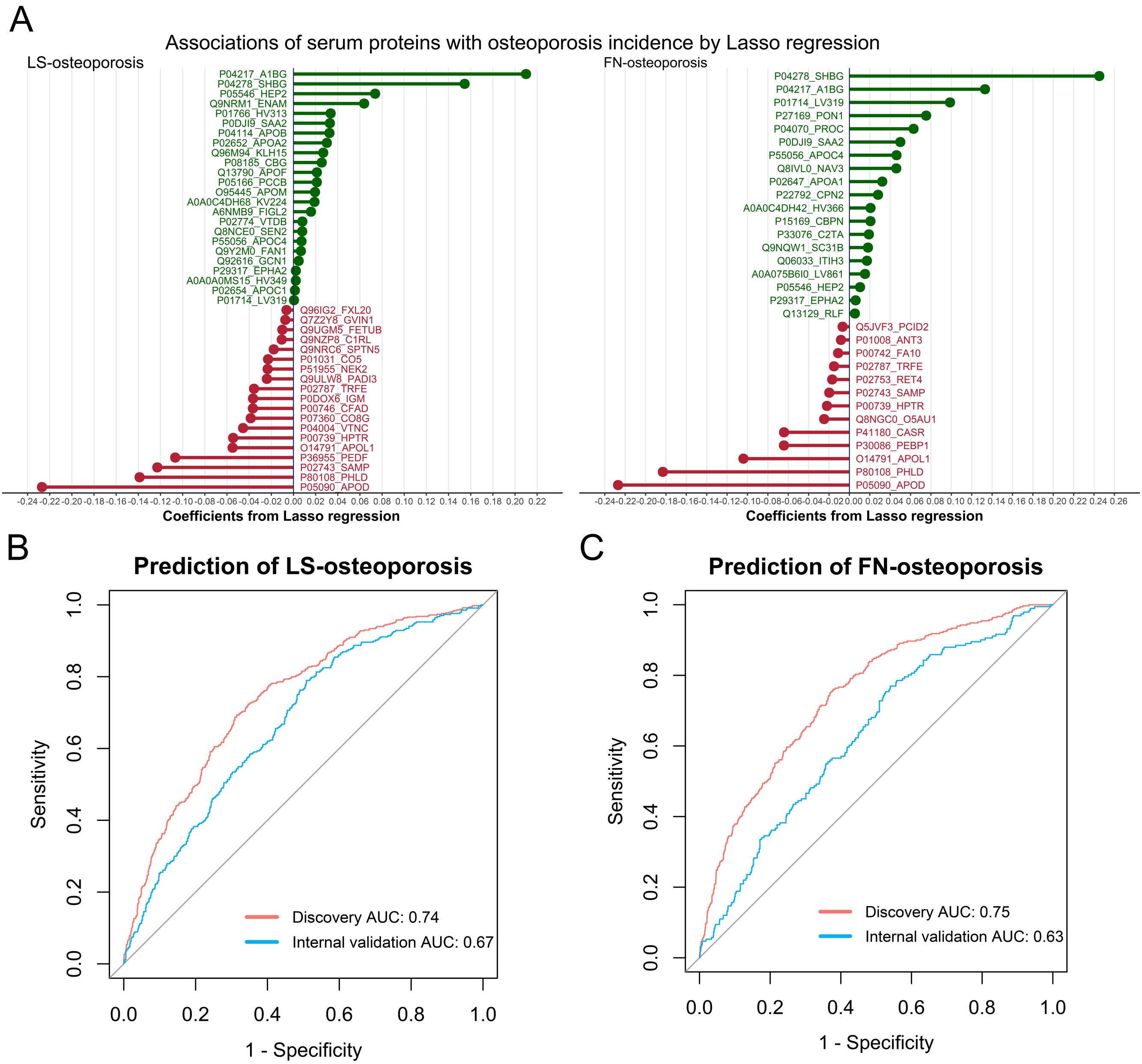

A
Associations of serum proteins with osteoporosis incidence by Lasso regression
LS-osteoporosis
P04217_A1BG
P04278_SHBG
P05546_HEP2
Q9NRM1_ENAM
P01766_HV313
P0DJI9_SAA2
P04114_APOB
P02652_APOA2
Q96M94_KLH15
P08185_CBG
Q13790_APOF
P05166_PCCB
O95445_APOM
A0A0C4DH68_KV224
A6NMB9_FIGL2
P02774_VTDB
Q8NCE0_SEN2
P55056_APOC4
Q9Y2M0_FAN1
Q92616_GCN1
P29317_EPHA2
A0A0A0MS15_HV349
P02654_APOC1
P01714_LV319
Q96IG2_FXL20
Q7Z2Y8_GVIN1
Q9UGM5_FETUB
Q9NZP8_C1RL
Q9NRC6_SPTN5
P01031_CO5
P51955_NEK2
Q9ULW8_PADI3
P02787_TRFE
P0DOX6_IGM
P00746_CFAD
P07360_CO8G
P04004_VTNC
P00739_HPTR
O14791_APOL1
P36955_PEDF
P02743_SAMP
P80108_PHLD
P05090_APOD
Coefficients from Lasso regression
FN-osteoporosis
P04278_SHBG
P04217_A1BG
P01714_LV319
P27169_PON1
P04070_PROC
P0DJI9_SAA2
P55056_APOC4
Q8IVL0_NAV3
P02647_APOA1
P22792_CPN2
A0A0C4DH42_HV366
P15169_CBPN
P33076_C2TA
Q9NQW1_SC31B
Q06033_ITIH3
A0A075B6I0_LV861
P05546_HEP2
P29317_EPHA2
Q13129_RLF
Q5JVF3_PCID2
P01008_ANT3
P00742_FA10
P02787_TRFE
P02753_RET4
P02743_SAMP
P00739_HPTR
Q8NGC0_O5AU1
P41180_CASR
P30086_PEBP1
O14791_APOL1
P80108_PHLD
P05090_APOD
Coefficients from Lasso regression
B
Prediction of LS-osteoporosis
Sensitivity
1 - Specificity
Discovery AUC: 0.74
Internal validation AUC: 0.67
C
Prediction of FN-osteoporosis
Sensitivity
1 - Specificity
Discovery AUC: 0.75
Internal validation AUC: 0.63
