# Supplementary material for: Proteome‐wide profiling reveals dysregulated molecular features and accelerated aging in osteoporosis: A 9.8‐year prospective study
Source: Aging Cell. 2023 Nov 16;23(2):e14035. doi: 10.1111/acel.14035 (PMC10861190; doi:10.1111/acel.14035)
Supplement: Supplementary file 1 — Figures S1–S10 [file ACEL-23-e14035-s002.zip › acel14035-sup-0005-FigureS5.pdf]

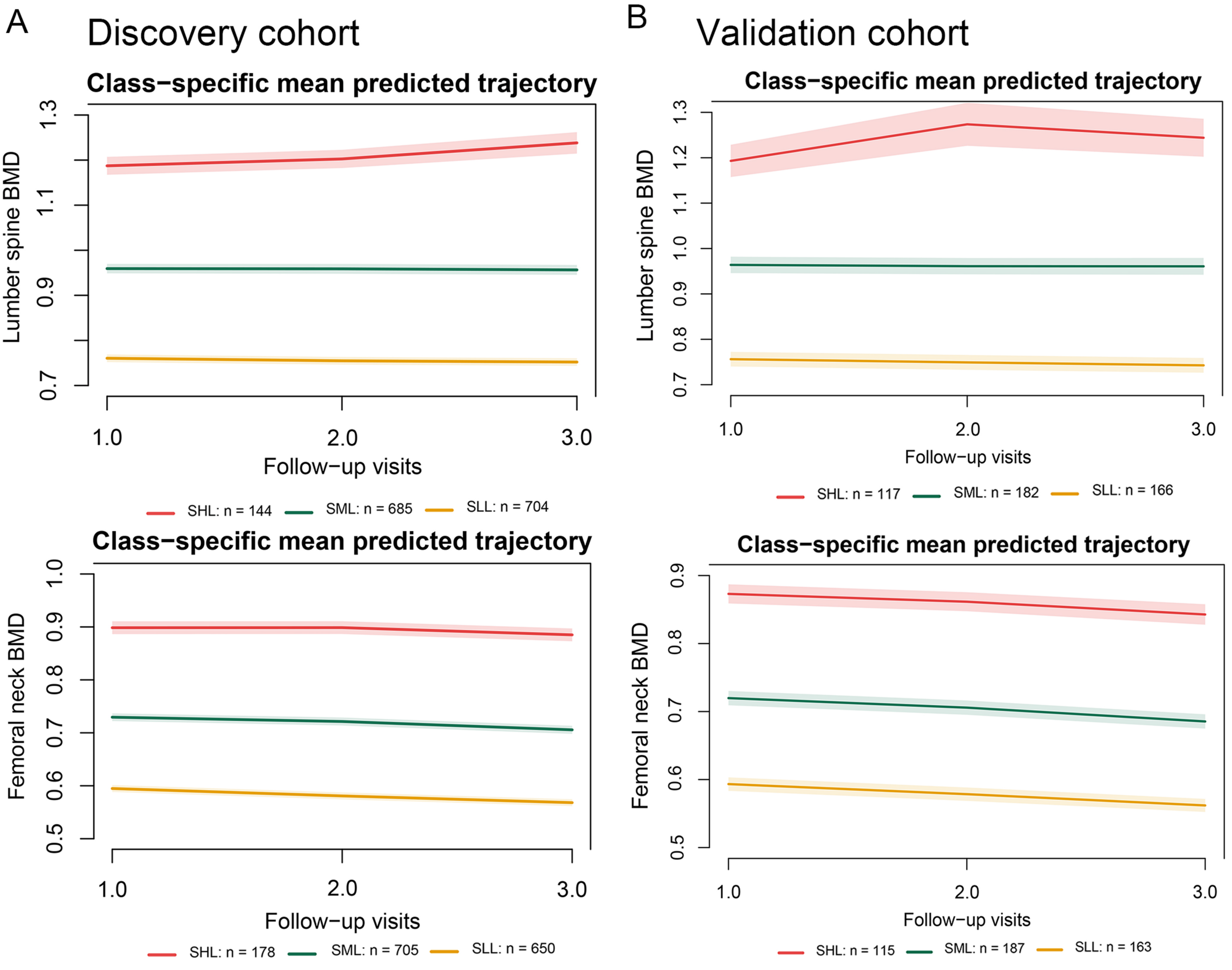

A
Discovery cohort
B
Validation cohort
Class−specific mean predicted trajectory
Lumber spine BMD
Femoral neck BMD
Follow−up visits
1.0
2.0
3.0
SHL: n = 144
SML: n = 685
SLL: n = 704
SHL: n = 117
SML: n = 182
SLL: n = 166
SHL: n = 178
SML: n = 705
SLL: n = 650
SHL: n = 115
SML: n = 187
SLL: n = 163
