# Supplementary material for: Proteome‐wide profiling reveals dysregulated molecular features and accelerated aging in osteoporosis: A 9.8‐year prospective study
Source: Aging Cell. 2023 Nov 16;23(2):e14035. doi: 10.1111/acel.14035 (PMC10861190; doi:10.1111/acel.14035)
Supplement: Supplementary file 1 — Figures S1–S10 [file ACEL-23-e14035-s002.zip › acel14035-sup-0006-FigureS6.pdf]

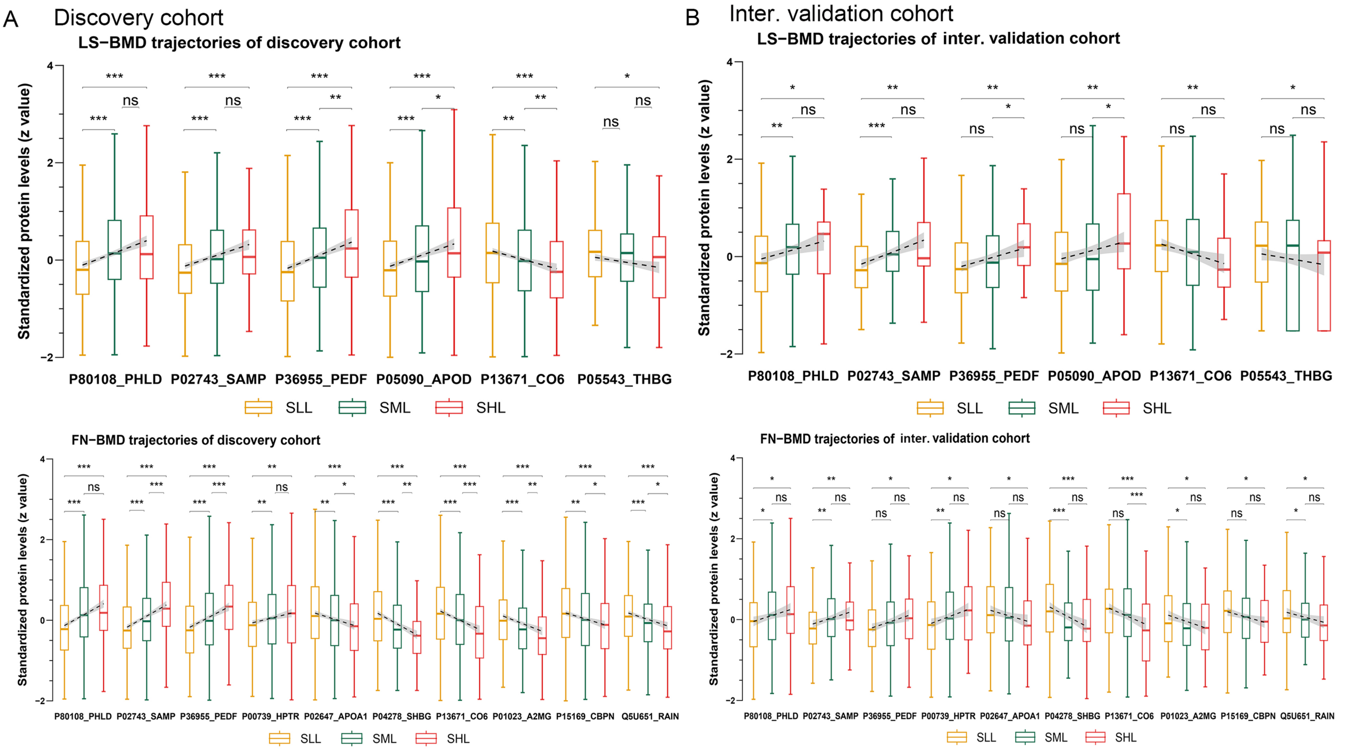
A
Discovery cohort
LS−BMD trajectories of discovery cohort
Standardized protein levels (z value)
P80108_PHLD
P02743_SAMP
P36955_PEDF
P05090_APOD
P13671_CO6
P05543_THBG
SLL
SML
SHL
FN−BMD trajectories of discovery cohort
P00739_HPTR
P02647_APOA1
P04278_SHBG
P01023_A2MG
P15169_CBPN
Q5U651_RAIN
B
Inter. validation cohort
LS−BMD trajectories of inter. validation cohort
FN−BMD trajectories of inter. validation cohort
