# Supplementary material for: Proteome‐wide profiling reveals dysregulated molecular features and accelerated aging in osteoporosis: A 9.8‐year prospective study
Source: Aging Cell. 2023 Nov 16;23(2):e14035. doi: 10.1111/acel.14035 (PMC10861190; doi:10.1111/acel.14035)
Supplement: Supplementary file 1 — Figures S1–S10 [file ACEL-23-e14035-s002.zip › acel14035-sup-0007-FigureS7.pdf]

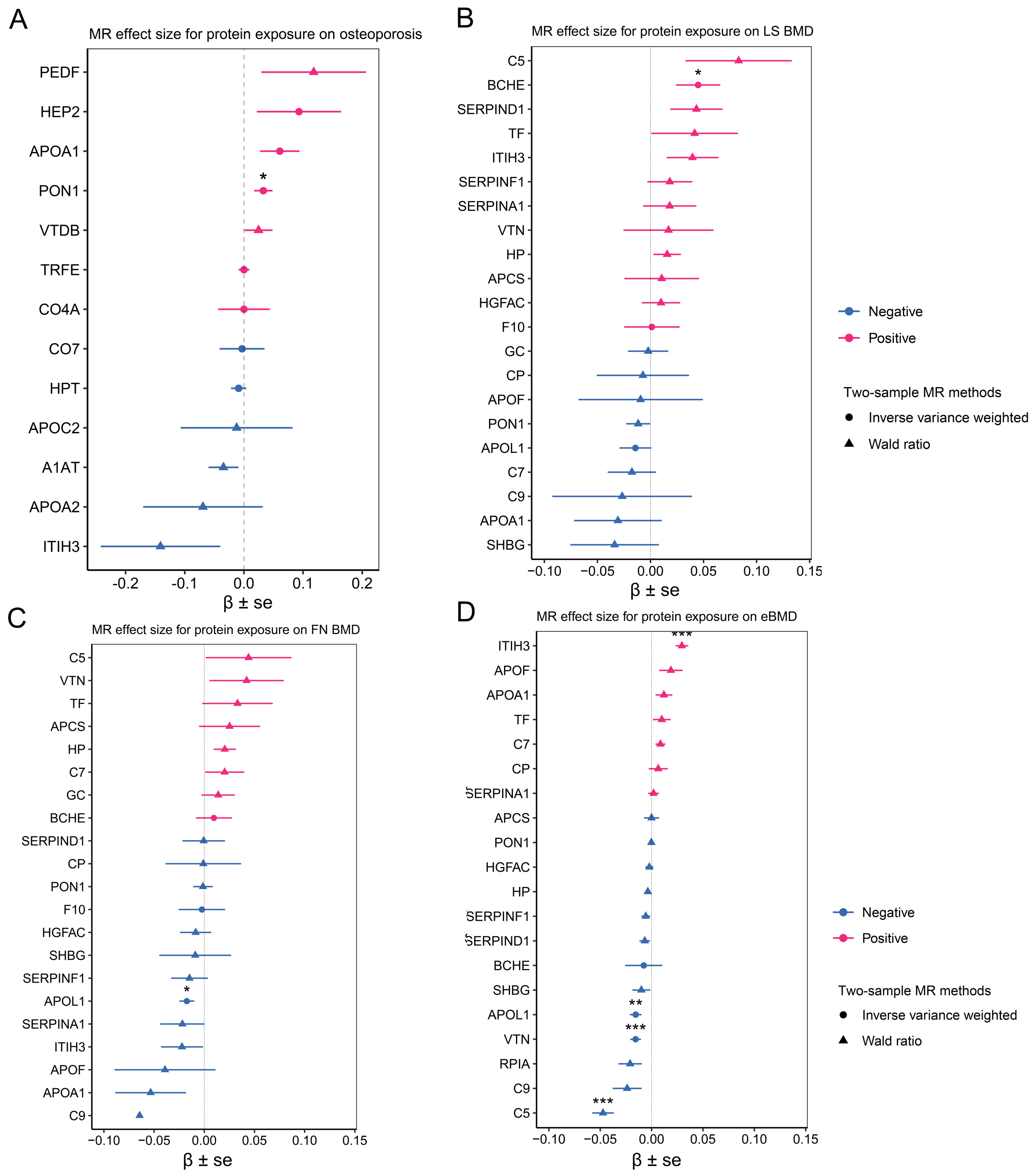

A
MR effect size for protein exposure on osteoporosis
PEDF
HEP2
APOA1
PON1
VTDB
TRFE
CO4A
CO7
HPT
APOC2
A1AT
APOA2
ITIH3
-0.2
-0.1
0.0
0.1
0.2
β ± se
B
MR effect size for protein exposure on LS BMD
C5
BCHE
SERPIND1
TF
ITIH3
SERPINF1
SERPINA1
VTN
HP
APCS
HGFAC
F10
GC
CP
APOF
PON1
APOL1
C7
C9
APOA1
SHBG
−0.10
−0.05
0.00
0.05
0.10
0.15
β ± se
Negative
Positive
Two-sample MR methods
Inverse variance weighted
Wald ratio
C
MR effect size for protein exposure on FN BMD
C5
VTN
TF
APCS
HP
C7
GC
BCHE
SERPIND1
CP
PON1
F10
HGFAC
SHBG
SERPINF1
APOL1
SERPINA1
ITIH3
APOF
APOA1
C9
−0.10
−0.05
0.00
0.05
0.10
0.15
β ± se
D
MR effect size for protein exposure on eBMD
ITIH3
APOF
APOA1
TF
C7
CP
SERPINA1
APCS
PON1
HGFAC
HP
SERPINF1
SERPIND1
BCHE
SHBG
APOL1
VTN
RPIA
C9
C5
−0.10
−0.05
0.00
0.05
0.10
0.15
β ± se
Negative
Positive
Two-sample MR methods
Inverse variance weighted
Wald ratio
