# Supplementary material for: Proteome‐wide profiling reveals dysregulated molecular features and accelerated aging in osteoporosis: A 9.8‐year prospective study
Source: Aging Cell. 2023 Nov 16;23(2):e14035. doi: 10.1111/acel.14035 (PMC10861190; doi:10.1111/acel.14035)
Supplement: Supplementary file 1 — Figures S1–S10 [file ACEL-23-e14035-s002.zip › Figure captions.docx]

Figure S1: The longitudinal change of BMD.
The change of BMD during the follow-up periods. The BMD levels were repeatedly scanned from the first to the third follow-up visits within 6.6 follow-up years, and 1974 participants possessed all measurements at three follow-up visits. The p value <0.05 was considered as statistically significant. ***p < 0.001, **p < 0.01, *p < 0.05.

Figure S2: Machine learning model for predicting the risk of osteoporosis.
A. Longitudinal serum proteomic profiles in discovery and internal validation cohorts. B. Workflow of the machine learning model built with quantitative proteomics data and clinical features. The population was separated into discovery and internal validation cohorts based on the proteomics test batches. (the first batch was discovery cohort, n = 1785), and the second batch was internal validation cohort, n = 1630). During the 9.8 follow-up years, 2966 people were tracked, with 1746 (LS-OP = 499, FN-OP = 330) in the discovery cohort and 1220 (LS-OP = 337, FN-OP = 191) in the internal validation cohort. We performed dimensionality reduction analysis of the proteome and identified proteomic features associated with osteoporosis using LightGBM and Lasso regression models. The LightGBM model discovered 38 LS-OP proteins and 28 FN-OP proteins in the two investigations, with 13 proteins overlapping, and the Lasso model uncovered 43 LS-OP proteins and 32 FN-OP proteins, with 13 proteins overlapping. Abbreviations: FN, femoral neck; LS, lumbar spine; OP, osteoporosis; LightGBM, light gradient boosting machine; Lasso, least absolute shrinkage and selection operator; ROC, receiver operating characteristic.

Figure S3: Identification of serum proteomic features for osteoporosis by LightGBM model. Shapley importance of proteomic features for LS-OP (A) and FN-OP (B). Receiver operating characteristic (ROC) plot of LightGBM model for LS-OP (C) and FN-OP (D). The LightGBM algorithm was used to create a machine learning model from 1746 samples including 314 serum proteins in the discovery cohort (training and testing datasets), and the model was validated in the internal validation cohort with 1220 samples. We optimized our model using a ten-fold cross internal validation. Next, we tested the model using the randomly selected testing dataset. Abbreviations: FN, femoral neck; LS, lumbar spine; OP, osteoporosis; LightGBM, light gradient boosting machine; ROC, receiver operating characteristic.

Figure S4: Identification of serum proteomic features for osteoporosis by LASSO model. The identified proteomic features for LS-OP and FN-OP in LASSO model (A). Receiver operating characteristic (ROC) plot of LASSO model for LS-OP (B) and FN-OP (C). The LASSO regression was used to create a machine learning model from 1746 samples including 314 serum proteins in the discovery cohort, and the model was validated in the internal validation cohort with 1220 samples. We optimized our model using a ten-fold cross internal validation. Abbreviations: FN, femoral neck; LS, lumbar spine; OP, osteoporosis; Lasso, least absolute shrinkage and selection operator; ROC, receiver operating characteristic.

Figure S5: The longitudinal trajectories of BMD.
The longitudinal trajectories of BMD in the discovery (A) and internal validation (B) cohorts. The latent class trajectory model (LCTM) was used to generate the latent trajectories of BMD levels at LS and FN across three follow-up visits. Sustained high level (SHL), sustained medium level (SML), and sustained low level (SLL) are three different trajectories. The minimum Bayesian information criterion (BIC) was used to confirm the best model and number of groups. Abbreviations: BMD, Bone mineral density; FN, Femoral neck; LS, Lumbar spine.

Figure S6: The different expression of proteins among the latent trajectories.
The different expression of important protein biomarkers among different latent trajectories of BMD in the discovery (A) and internal validation (B) cohorts. The Benjamini-Hochberg (BH) false discovery rates (FDR) approach was applied to control alpha error. ***FDR <0.001, ** FDR <0.01, * FDR <0.05.

Figure S7: The causal association between proteins and bone health.
A. The causal association between serum proteins and osteoporosis in East Asian population. B. The causal association between plasma proteins and LS-BMD in European population. C. The causal association between plasma proteins and FN-BMD in European population. D. The causal association between plasma proteins and estimated BMD at heel in European population. The causal effect of proteins on bone health was evaluated by two-sample MR analyses. The GWAS-summary data of circulatory proteins were extracted from Chinese (Xu et al., 2023) and European populations (Emilsson et al., 2018; Suhre et al., 2017; Sun et al., 2018; Yao et al., 2018). The GWAS-summary data of osteoporosis were extracted from Japanese (Ishigaki et al., 2020). The summary SNP-BMD coefficients for LS and FN were extracted from the GWAS study in European populations (Zheng et al., 2015). The GWAS summary data of eBMD were estimated by heel quantitative ultrasound in UK biobank (Al-Ansari et al., 2022). All the effect size and standard error of osteoporosis and BMDs were extracted from three GWAS studies (osteoporosis, cases/controls = 7788/204,665; FN-BMD, n = 32,735; LS-BMD, n = 28,498; eBMD, n = 426,824). The MR Egger, Weighted median, Inverse variance weighted, Simple mode, and Weighted mode were performed for multiple genetic SNPs, and Wald ratio was performed for single SNP. Abbreviations: BMD, bone mineral density; FN, femoral neck; LS, lumbar spine; MR, Mendelian randomization; SNP, single nucleotide polymorphism.

Figure S8: Biological age of bone constructed from proteomics data.
A. Association of BMD-proteins with chronological age. B. Relationships between biological age scores and chronological age (CA) and their linear regression lines. The bone biological age (BA) was proposed by Klemera and Doubal algorithm based on the identified proteomic features. The construction of BA was conducted for male and female groups separately. Each feature was regressed into CA and only features significantly correlated with age (p < 0.05) were utilized for the generation of BA. Moreover, features with redundant information were filtered out. The difference between KDM-Proage and chronological age was defined as biological age acceleration (BioAgeAccel). The biological age scores including KDM-Proage (Klemera and Doubal proteomics age) and BioAgeAccel.

Figure S9: Flowchart of inclusion criteria for participants in this study
Totally, 4048 participants eligible for inclusion in the Guangzhou Nutrition and Health Study (GNHS) at baseline. The exclusion criteria for participants in further study were: (1) Missing important variables (age, sex and so on) at baseline and missing BMD measures at 1st follow-up: 206; (2) Patients with history of fracture and disease that affects BMD at baseline (hyperthyroidism, cancer and uremia): 337; (3) Without proteomics data at baseline: 261. Finally, 3244 participants at baseline were retained for further study.

Figure S10: The hypothesis of mendelian randomization analysis.
The two-sample mendelian randomization (MR) analysis with public summary data was used to validate the association of plasma protein with OP and BMD. The main assumptions of two-sample MR analysis were shown below: (1) the genetic instrumental variables are strongly associated with plasma proteins; (2) the genetic instrumental variables are not associated with any known or unmeasured confounders: influencing the association between genetic variants and outcomes; (3) the genetic variants are associated with outcomes only through exposures: variants causing significant effects on outcomes not through other pathways, no horizontal pleiotropy. Abbreviations: BMD, Bone mineral density; OP, Osteoporosis; FN, Femoral neck; LS, Lumbar spine; MR, Mendelian randomization; eBMD, Estimated BMD; SNP, Single nucleotide polymorphism.
