# Supplementary figures and images for: Proteome‐wide profiling reveals dysregulated molecular features and accelerated aging in osteoporosis: A 9.8‐year prospective study
Source: Aging Cell. 2023 Nov 16;23(2):e14035. doi: 10.1111/acel.14035 (PMC10861190; doi:10.1111/acel.14035)

## Lumbar spine

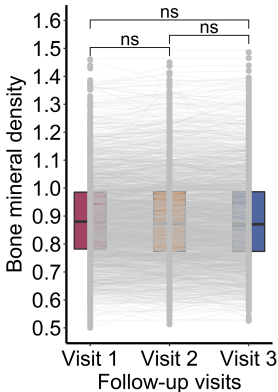

## Femoral neck

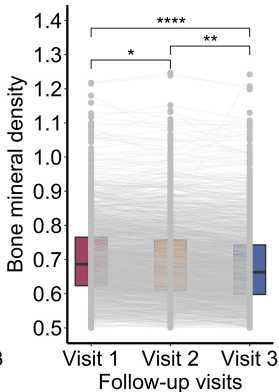

Supplement: Supplementary file 1 — Figures S1–S10 [file ACEL-23-e14035-s002.zip › acel14035-sup-0001-FigureS1.pdf]

A

## The Guangzhou Nutrition and Health study (GNHS)

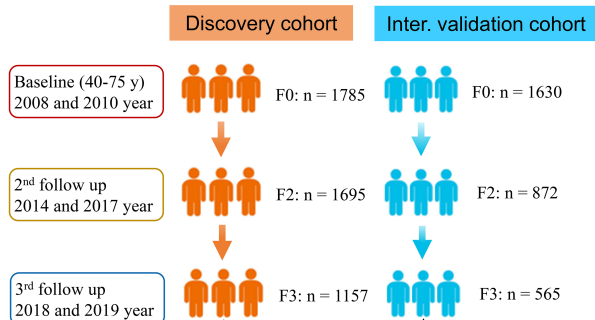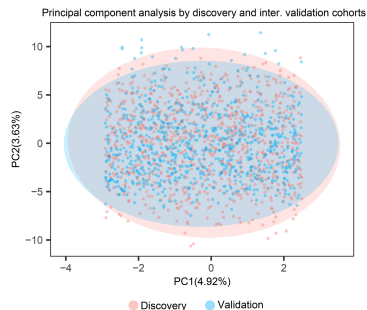

B

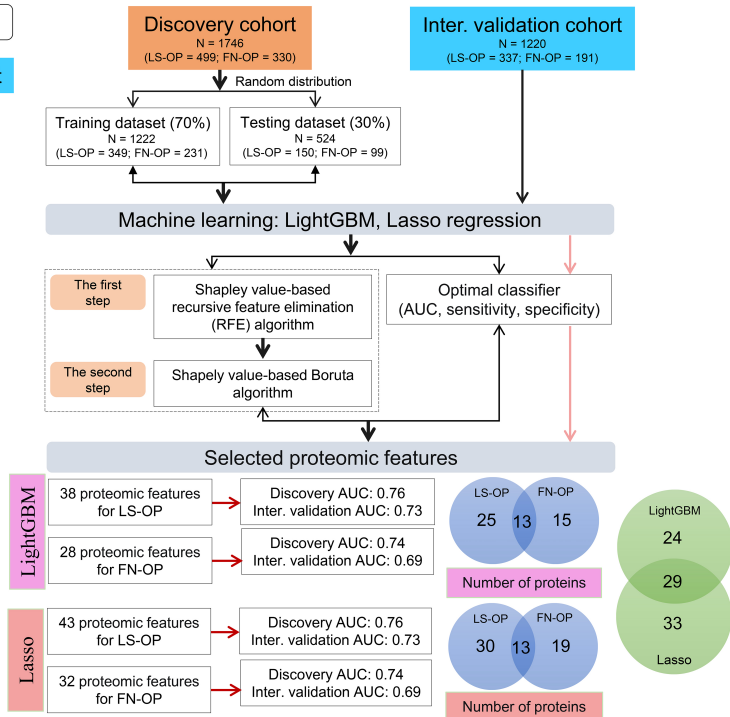

Supplement: Supplementary file 1 — Figures S1–S10 [file ACEL-23-e14035-s002.zip › acel14035-sup-0002-FigureS2.pdf]

A

Association of chronological age with proteins

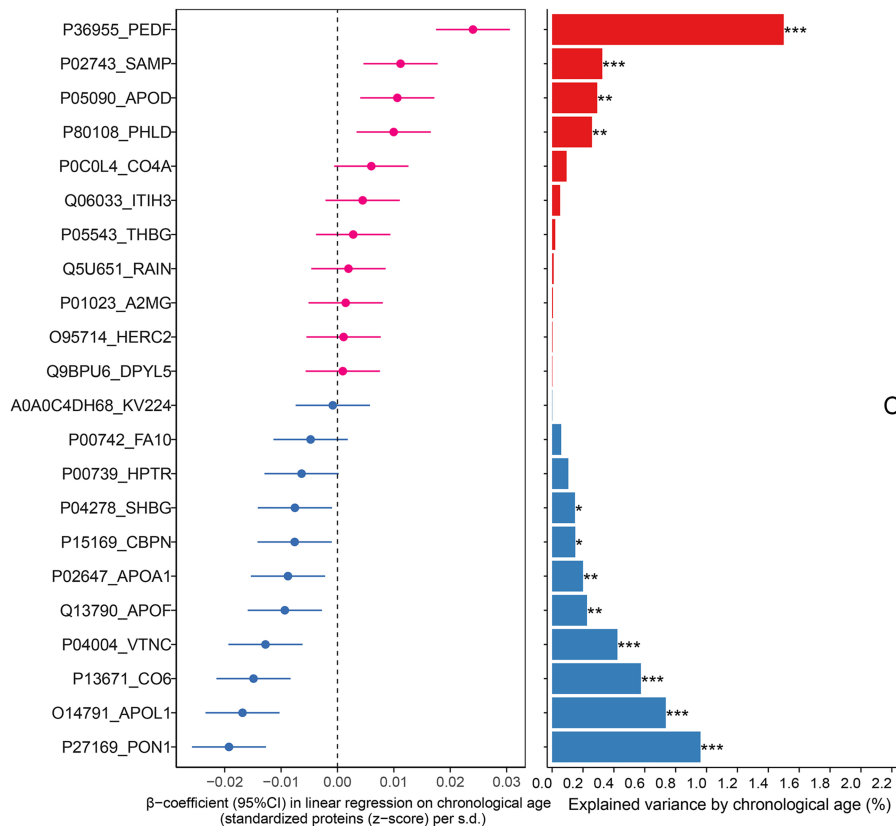

B

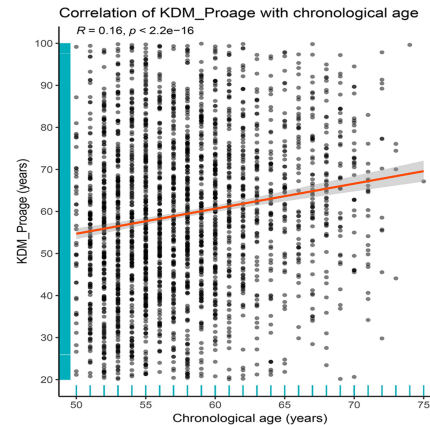

C

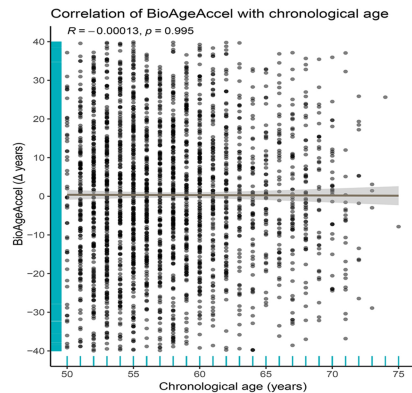

Supplement: Supplementary file 1 — Figures S1–S10 [file ACEL-23-e14035-s002.zip › acel14035-sup-0008-FigureS8.pdf]

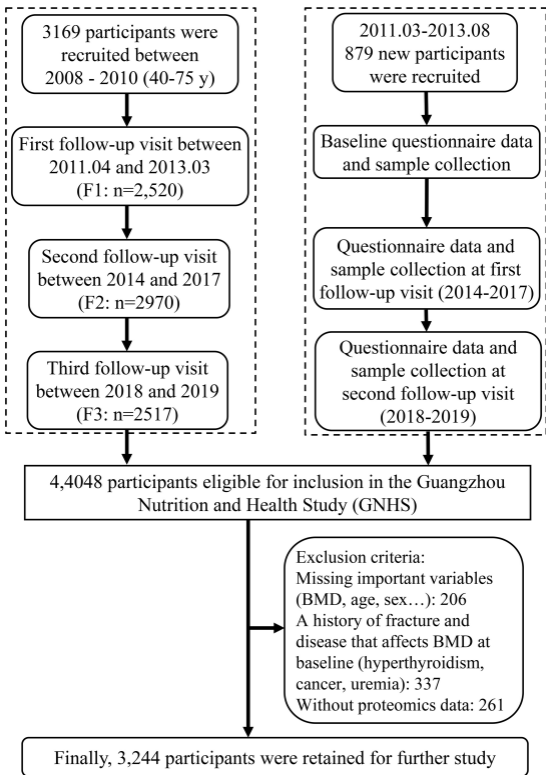

Supplement: Supplementary file 1 — Figures S1–S10 [file ACEL-23-e14035-s002.zip › acel14035-sup-0009-FigureS9.pdf]

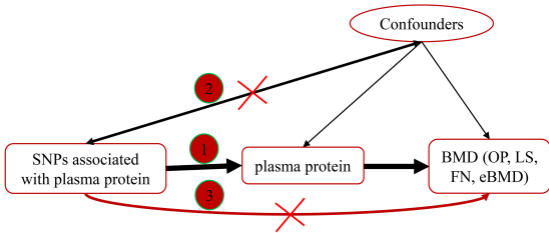

Supplement: Supplementary file 1 — Figures S1–S10 [file ACEL-23-e14035-s002.zip › acel14035-sup-0010-FigureS10.pdf]
