# Supplementary material for: Proteome‐wide profiling reveals dysregulated molecular features and accelerated aging in osteoporosis: A 9.8‐year prospective study
Source: Aging Cell. 2023 Nov 16;23(2):e14035. doi: 10.1111/acel.14035 (PMC10861190; doi:10.1111/acel.14035)
Supplement: Supplementary file 3 — Appendix S1 [file ACEL-23-e14035-s003.docx]

**Supplementary Materials**

**Supplemental Methods**

**Method S1 Assessment of covariates and laboratory measures**

The venous blood samples of eligible participants were collected after overnight fasting (over 12h), and serum was separated after centrifugation at 4°C within 2h and stored at −80°C for further analysis during the follow-up visit.

The information about medical history, socio-demographic characteristic, and habitual dietary intake was collected by face-to-face questionnaire and food frequency questionnaire at every study time points. Anthropometric measurements were measured by trained staff. The covariates included in this study was further categorized into 5 groups:

1. 4 demographic factors: age, sex, household income, and self-reported

educational level; (2) 9 lifestyle and dietary factors: physical activity, total energy intake, alcohol drinking, smoking, vegetable intake, fruit intake, fish intake, red and processed meat intake, and yogurt intake; (3) 5 blood test factors: Fasting glucose, HDL-c, LDL-c, TC, and TG; (4) 8 anthropometry factors: height, weight, hip circumference, waist circumference, neck circumference, BMI, DBP, SBP; (5) 12 medical history: hypertension, type 2 diabetes, dyslipidemia, cardiovascular disease, gastric ulcer, gout, fatty liver disease, menopause, estrogen supplement, use of anti-osteoporosis drug, calcium supplement, multi-vitamins supplement.

The socio-demographic factors, medical history, lifestyle factors and dietary information over the past 12 months were collected through face-to-face interviews by a validated 79-item food frequency questionnaire (FFQ) [1]. The metabolic equivalent for task (MET) hours per day (excluding sleeping and sitting) was calculated on the basis of a 19-item questionnaire for physical activity [2]. Height was measured to the nearest 0.5 cm, and weight to the nearest 0.1 kg in light clothing and without shoes. Body mass index (BMI) was calculated as weight (kg)/height (m)^2^. Waist circumference (WC) was measured half way between the lowest rib margin and the iliac crest, while hip circumference was measured at the level of the greater trochanters. Waist to hip ratio (WHR) was then calculated. Measurements of waist and hip circumference were performed twice to the nearest 0.5 cm and the mean of the two measurements were used for subsequent analysis. Two consecutive measurements of blood pressure were taken from right arm after each subject had been sitting for at least 10 min. Systolic blood pressure (SBP) and diastolic blood pressures (DBP) were recorded to the nearest 2 mm Hg. If the two systolic or diastolic blood pressures recorded were ≥4 mmHg apart, a third measurement was made. The average of the two blood pressures was used for the subsequent analysis. SBP and DBP were defined as the point of the appearance (Korotkoff I) and disappearance (Korotkoff V) of Korotkoff sounds, respectively. Moreover, education (middle school or lower, high school or professional college, university), alcohol drinking (current and non-current drinker), smoking (current and non-current smoker), household income (≤500, 501-1500, 1501-3000, >3000 Chinese Yuan/month/person) were defined.

Blood samples of all participants were collected after 12 h overnight fasting using vacuum tubes containing EDTA. Plasma was separated after centrifugation at 1500 × g for 15 min at 4 °C within 2 h and stored at -80 °C till tests. All samples were analyzed in a single batch within 5 days to minimize laboratory variability. Plasma total cholesterol (TC), triglycerides (TG), HDL cholesterol (HDL-C) and LDL cholesterol (LDL-C) were measured with colorimetric methods using commercial kits (Biosino Biotechnology Company Ltd, Beijing, China) by an automated analyzer (A25 Biosystem, Barcelona, Spain). The coefficient of variation for lipid measurements was 2.3% (at 4.50 mmol/L TC), 5.8% (at 1.77 mmol/L TG), 4.3% (at 1.28 mmol/L HDL-C), and 3.1% (at 3.29 mmol/L LDL-C). Colorimetric methods were performed with a Hitachi 7600-010 automated analyzer to measure the fasting serum glucose.[3] Laboratory assay was conducted by two staff who were not involved in the questionnaire interview or did not know the relevant exposure status of the subjects.

**Method S2 Serum proteome profiling**

The serum was prepared using a standardized protocol and stored in 0.5 ml aliquots at -80°C. 1 μL of a serum sample from each participant was analyzed using MS-based proteomics technology. The serum was firstly denatured with 20 µL of lysis buffer containing 8 M urea (Sigma, #U1230) in 100 mM ammonium bicarbonate (ABB) at 30°C for 30 min. The lysates were reduced with 10 mM tris (2-carboxyethyl) phosphine (TCEP, Sigma #T4708) at 30°C for 30 min, and were then alkylated with 40 mM iodoacetamide (IAA, Sigma, #SLCD4031) in darkness for 45 min. The solution was then diluted with 70 µL 100 mM ABB to make sure urea concentration was less than 1.6M and was subjected to 2-step overnight tryptic digestion (Hualishi Tech. Ltd, Beijing, China), at an enzyme/substrate ratio of 1:60 for 4 h and 12 h, successively. Thereafter, the digestion was quenched with 1% trifluoroacetic (Thermo Fisher Scientific, #T/3258/PB05) to pH 2-3. Peptides were cleaned using C18 (Thermo, #60209-001).

Peptide samples were then analyzed by LC-MS/MS on a TripleTOF 5600 system (SCIEX, CA, USA) coupled to Eksigent NanoLC 400 System (Eksigent, Dublin, CA, USA). Briefly, peptides were loaded onto a trap column (5 µm, 120 Å, 10 × 0.3 mm), and were separated along a 20 min linear LC gradient from 5% to 32% buffer B (buffer B: 98% ACN, 0.1% formic acid in HPLC water; buffer A: 2% ACN, 0.1% formic acid in HPLC water) on an analytical column (3 µm, 120 Å, 150 × 0.3 mm) at a flow rate of 5 µL/min. The SWATH-MS method is composed of a 100 ms of full TOF MS scan with the acquisition range of 350-1250 *m/z*, followed by 55 sequential MS/MS scans of variable m/z isolation windows from 100 to 1500 Da. The accumulation time was set at 30 ms per isolation window, resulting in a total cycle time of 1.9 s.

After SWATH acquisition, the wiff files were analyzed using DIA-NN (1.7.12) against a serum spectral library containing 3,474 peptide precursors and 536 unique proteins from Swiss-Prot database of Homo Sapiens. In the DIA-NN setting, the peptide length range was set from 5 to 30, the precursor m/z range was set from 400 to 1200, and the fragment ion m/z range was set from 100 to 1500. The retention time extraction window was automatically set by the software, and the m/z extraction window for MS1 and MS2 was 20 ppm and 50 ppm, respectively. Protein and peptide FDRs were controlled below 1%. PyProphet (version 0.24) was used to control FDR<0.01 at both peak group and peptide level by target-decoy strategy. Peptide matrix from PyProphet was converted to protein matrix using ProteomeExpert. In brief, we sorted peptides in each protein first by its missing rate and then by mean intensity. Proteins’ intensity was determined by the mean intensity of its top 3 unique peptides. In cases no more than 3 peptides were identified, all the peptides per protein were used for protein intensity computation. For the Skyline analysis, the retention time was predicted by the CiRT peptides mentioned above, and the isolation time window was set as 2 min. The mass analyzer for MS1 and MS/MS was set as “TOF” with a resolution power of 30,000.

**Method S3 Quality control of proteome data**

The quality of proteomic data was ensured at multiple levels. First, serum samples were randomly distributed in 58 different batches. Every batch contains a sample-preparation QC sample made from mixture of several serum samples, and a peptide pool sample for monitoring instrument state, respectively. Additional analyses of technical and biological replicates were performed to evaluate the reproducibility of the data. The median coefficients of variance (CV) of QC samples for sample-preparation and LC-MS performance are 2.4% and 2%, respectively. The median spearman correlation within the technical and biological replicates were 0.99 and 0.91 respectively.

**Method S4 BMD proteins risk score**

We used the identified site-specific proteomic biomarkers in this study to construct a protein risk score (PRS) as

$${PRS}_{i}=\sum_{j=1}^{n} \beta_{j}Z_{ij}$$

Where,${PRS}_{i}$ is a site-specific protein risk score for individual *i,* $n$ is the number of the identified proteomic biomarkers for BMD, $Z_{ij}$is the standardized abundance (Z score) of the protein *j* for individual *i*. $\beta_{j}$is the regression coefficients in meta-analysis for continuous BMD for protein *j* after standardization in baseline.

**Method S5 The selection criteria of genetic instruments for proteins**

The summarizes of protein genetic instrument were selected from the large-scale GWAS with standardize criteria were shown below:

Firstly, the strongest variants (pQTL) for each protein were used as the instrumental variable (using a P-value threshold ≤5 × 10^-8^), including both cis and trans pQTLs [4]. The MR Egger, weighted median, simple mode, weighted mode, and inverse variance weighted, Wald ratio methods were used to obtain two-sample MR effect estimates [5-7]. In this analysis, the MR effect estimates were sensitive to the particular choice of pQTLs, since only the most strongly associated SNPs within each genomic region were used as instruments. Due to the complex LD structure of SNPs within the human Major Histocompatibility Complex (MHC) region, we removed SNPs and proteins coded by genes within the MHC region (chr6: from 26Mb to 34Mb) [8]. We then conducted linkage disequilibrium (LD) clumping for the instruments with the TwoSampleMR R package to identify independent pQTLs for each protein. We used r^2^< 0.001 as the threshold to exclude dependent pQTLs in the cis (or trans) gene region.

**Method S6 Calculation of Biological Age**

Klemera and Doubal developed a mathematical model (KDM) that estimates biological age based on selected proteins that correlate with the chronological age (CA) for any sex [9].

$$KDM-Proage=\frac{\sum_{j=1}^{m} \left( x_{j}-q_{j} \right)\left( \frac{k_{j}}{s_{j}^{2}} \right)+\frac{\mathrm{CA}}{s_{\mathrm{BA}}^{2}}}{\sum_{j=1}^{m} \left( \frac{k_{j}}{s_{j}^{2}} \right)+\frac{1}{s_{\mathrm{BA}}^{2}}}$$

The values *k*, *q*, and *s* are the regression slope, intercept, and the root means squared error of a biomarker regressed on chronological age, respectively.

**References**

1. Zhang CX, Ho SC: Validity and reproducibility of a food frequency Questionnaire among Chinese women in Guangdong province. Asia Pac J Clin Nutr. 2009;18(2):240-250.

2. Liu B, Woo J, Tang N, Ng K, Ip R, Yu A: Assessment of total energy expenditure in a Chinese population by a physical activity questionnaire: examination of validity. International journal of food sciences and nutrition. 2001;52(3):269-282.

3. Zhang B, Chen YM, Huang LL, Zhou XX, Chen CG, Ye YB, Su YX: Greater habitual soyfood consumption is associated with decreased carotid intima-media thickness and better plasma lipids in Chinese middle-aged adults. Atherosclerosis. 2008;198(2):403-411.

4. Tin A, Köttgen A: Mendelian Randomization Analysis as a Tool to Gain Insights into Causes of Diseases: A Primer. J Am Soc Nephrol. 2021;32(10):2400-2407.

5. Burgess S, Thompson SG: Interpreting findings from Mendelian randomization using the MR-Egger method. European journal of epidemiology. 2017;32(5):377-389.

6. Burgess S, Bowden J, Fall T, Ingelsson E, Thompson SG: Sensitivity Analyses for Robust Causal Inference from Mendelian Randomization Analyses with Multiple Genetic Variants. Epidemiology (Cambridge, Mass). 2017;28(1):30-42.

7. Burgess S, Scott RA, Timpson NJ, Davey Smith G, Thompson SG: Using published data in Mendelian randomization: a blueprint for efficient identification of causal risk factors. European journal of epidemiology. 2015;30(7):543-552.

8. Gazal S, Finucane HK, Furlotte NA, Loh PR, Palamara PF, Liu X, Schoech A, Bulik-Sullivan B, Neale BM, Gusev A *et al*: Linkage disequilibrium-dependent architecture of human complex traits shows action of negative selection. Nat Genet. 2017;49(10):1421-1427.

9. Klemera P, Doubal S: A new approach to the concept and computation of biological age. Mechanisms of ageing and development. 2006;127(3):240-248.
